# Modulation of the RNA‐sensing RIG/MAVS pathway bypasses resistance to hypomethylating agents in acute myeloid leukemia

**DOI:** 10.1002/hem3.70523

**Published:** 2026-11-11

**Authors:** Nadia El Khawanky, Tatiana Nedelko, Giada Mandracci, Yunus Barth, Yuan Jiang, Kai Wang, Marc Rosenbaum, Christin Weiß, Christoph Geisenberger, Stefan Enßle, Ana M. Gómez Martínez, Sascha Göttert, Noomen Hamed, Julius C. Fischer, Jürgen Ruland, Dirk H. Busch, Hendrik Poeck, Katharina S. Götze, Anna L. Illert, Florian Bassermann, Simon M. Heidegger

**Affiliations:** ^1^ Department of Medicine III TUM School of Medicine and Health, TUM University Hospital, Technical University of Munich Munich Germany; ^2^ TranslaTUM Center for Translational Cancer Research, Technical University of Munich Munich Germany; ^3^ Institute of Clinical Chemistry and Patho‐Biochemistry, School of Medicine Technical University of Munich Munich Germany; ^4^ Institute of Pathology Ludwig‐Maximilians‐Universität München Munich Germany; ^5^ German Cancer Consortium (DKTK), Partner Site Munich, a Partnership Between DKFZ and University Hospital Munich Munich Germany; ^6^ Department of Internal Medicine III University Hospital Regensburg Regensburg Germany; ^7^ Institute of Medical Microbiology, Immunology, and Hygiene, TUM School of Medicine and Health Technical University of Munich Munich Germany; ^8^ Department of Radiation Oncology TUM School of Medicine and Health, TUM University Hospital, Technical University of Munich Munich Germany; ^9^ German Cancer Consortium (DKTK) Partner‐Site Munich and German Cancer Research Center (DKFZ) Heidelberg Germany; ^10^ Leibniz Institute for Immunotherapy (LIT) Regensburg Germany; ^11^ Center for Immunomedicine in Transplantation and Oncology (CITO) Regensburg Germany; ^12^ Bavarian Cancer Research Center (BZKF) Munich Germany; ^13^ Department of Hematology and Medical Oncology University Medical Center Göttingen (UMG) Göttingen Germany; ^14^ Present address: Early Clinical Development Oncology Genentech Inc. South San Francisco California United States

## Abstract

DNA hypomethylating agents (HMAs) such as 5‐azacytidine (5‐AZA) are a cornerstone in the treatment of acute myeloid leukemia (AML), but development of resistance represents a major clinical challenge. Apart from epigenetic reprogramming of malignant cell clones, therapy with HMA is increasingly recognized to facilitate antineoplastic immunity, yet the relevance of these molecular mechanisms in AML remains poorly defined. Here, we show that AML cell‐intrinsic sensing of aberrant RNA via the innate receptor system retinoic acid‐inducible gene I (RIG‐I)/mitochondrial antiviral‐signaling protein (MAVS) is essential for the therapeutic activity of HMAs. In human and murine AML cell lines, HMA treatment induced robust RIG‐I/MAVS pathway activation and subsequent Type I interferon (IFN‐I) release. HMA enhanced transcriptional activity of endogenous retroviral (ERV) elements that readily bound to the RIG‐I receptor. Analysis of a small series of patient samples linked treatment response to elevated gene expression of ERV and RIG‐I/MAVS pathway components in CD34^+^ cells including AML blasts. AML‐intrinsic genetic ablation of MAVS or RIG‐I abrogated the HMA treatment effects in mice by interfering with host IFN‐I signaling and subsequent expansion and accumulation of activated T cells. Acquired HMA resistance was associated with ERV suppression and blunted RIG‐I/MAVS signaling. Treatment with all*‐trans* retinoic acid (ATRA) restored RIG‐I pathway activity and antileukemic response in mice bearing HMA‐resistant AML. Our findings not only delineate a critical link between epigenetic modulation and innate immune activation in AML but also provide a rationale for targeting the RIG‐I/MAVS RNA‐sensing pathway to enhance antileukemic immunity and bypass epigenetic resistance to HMA.

## Graphical abstract

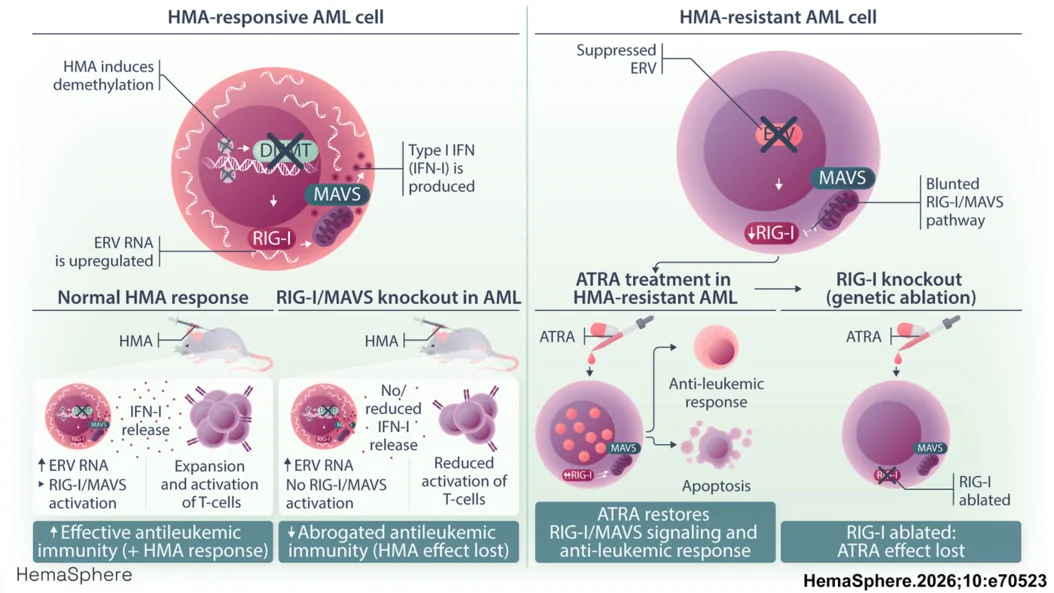

## INTRODUCTION

HMAs, such as 5‐azacytidine (5‐AZA), are a critical drug class in the treatment of acute myeloid leukemia (AML). Particularly in elderly patients, HMA and combination therapies are standard of care. However, resistance remains a major clinical challenge. HMAs are epigenetic drugs that introduce diverse modifications to histone proteins and nucleic acids that cooperatively regulate chromatin structure and ultimately gene expression.^1^ By incorporating into DNA or RNA, HMAs inhibit DNA methyltransferases, thereby reversing aberrant methylation patterns and reactivating, otherwise, silenced genes (e.g., tumor suppressors) within malignant cells.^2^ Beyond their direct antineoplastic effects—apoptosis induction, cell‐cycle arrest, and differentiation—HMAs are increasingly recognized to have immunostimulatory potential.^3^, ^4^, ^5^, ^6^

DNA methylation is widely used in eukaryotic cells to repress transcription of transposable elements, including endogenous retroviruses (ERVs) in the host genome.^7^ Re‐expression of ERVs upon HMA treatment has been linked to innate antiviral‐like immune responses within tumor cells.^8^, ^9^, ^10^ Aberrant ERV nucleic acids—potentially produced by bidirectional or reverse transcription and sense/antisense pairing—can be detected by innate nucleic acid sensors, including RIG‐I–like helicases (RLHs) and potentially cyclic GMP‐AMP synthase (cGAS).^9^, ^10^, ^11^ Activation of these receptors in tumor cells may initiate Type I interferon (IFN‐I) signaling, thereby triggering innate and adaptive immune responses. In stark contrast to actual clinical use of HMA, which is restricted to hematologic malignancies, most of these mechanistic findings stem from preclinical solid tumor models. Yet, emerging translational data mainly from patients with myelodysplastic syndrome (MDS) or chronic myelomonocytic leukemia (CMML) imply HMA treatment in myeloid malignancies may indeed lead to ERV reactivation and engagement of interferon and inflammatory programs.^12^ Herein, ERVs have been suggested to constitute a potential reservoir of T cell targets in hematological malignancies.^13^, ^14^ Still, the translatability and, hence, the relevance of HMA immunomodulation effects for the treatment of AML remained unclear.

The RLH receptor family—named after its eponymous member retinoic acid‐inducible gene I (RIG‐I)—detects foreign or mis‐localized endogenous cytosolic RNA.^15^ Upon engagement, RIG‐I or the family's second receptor MDA5 (melanoma differentiation antigen 5) recruits the adapter molecule MAVS (mitochondrial antiviral‐signaling protein), inducing complex innate immune transcriptional programs, including the release of IFN‐I (via the transcription factors IRF3/7 and NF‐Κb) and pro‐inflammatory cytokines. cGAS binds double‐stranded cytosolic DNA and then activates the adaptor protein STING (stimulator of IFN genes) for similar downstream signaling.^16^ In addition, tumor cell‐intrinsic nucleic acid receptor activation can trigger strong, cell‐autonomous apoptotic death.^17^, ^18^ Initially identified for their roles in antiviral defense, the RIG‐I/MAVS and cGAS/STING pathways are increasingly recognized for their involvement in the development of anti‐tumor T cell immunity.^19^, ^20^, ^21^

In this study, we show that HMA‐induced aberrant RNA expression and subsequent RIG‐I–mediated IFN‐I release by AML cells is indeed critical for the development of antileukemic T cell immunity and subsequent AML control. Furthermore, we provide evidence that modulation of the RIG‐I/MAVS pathway is a promising strategy to bypass HMA resistance in AML.

## METHODS

### Syngeneic AML mouse models

C57BL/6j and BALB/c mice were inoculated intravenously (i.v.) on Day 0 with 1 × 10^6^ C1498 or WEHI‐3B AML cells (in 100 µL phosphate buffered saline [PBS]), respectively. From Day 3, mice received intraperitoneal (i.p.) injections of either PBS or 5‐AZA (2.5 mg/kg in 100 µL) from Days 3 to 8 and 14 to 19, reflecting clinical dosing schedules. For immune cell depletion, mice were treated i.p. with anti‐CD4 (GK1.5), anti‐CD8 (YTS169.4), anti‐NK1.1 (PK136), or isotype control antibodies on Days −3 and −1 (200 µg), and again on Days 7 and 14 (100 µg) to maintain depletion. On Day 20, selected mice were sacrificed for flow cytometry of blood and bone marrow (BM); the rest were monitored until Day 60 for survival. Euthanasia criteria included hind limb paralysis, subcutaneous tumors (>2 cm), anemia, or health deterioration per animal welfare guidelines.

### CRISPR‐Cas–mediated genome editing of murine leukemia cell lines

CRISPR‐Cas9 was used to generate C1498 and WEHI‐3B cells deficient in Ddx58 (RIG‐I), Tmem173 (STING), or Mavs. Single guide RNAs (sgRNAs) were designed using CHOPCHOP or Benchling (Supporting Information S2: Table 2), complexed with TrueCut Cas9 Protein v2 at a 3:1 ratio, and delivered via electroporation (Lonza SF Kit, Program CM‐137) with electroporation enhancer and IDTE buffer. Knockouts were validated by immunoblotting and Sanger sequencing (Supporting Information S2: Table 2), with a ≥95% knockout score required for functional studies.

### Murine leukemia virus endogenous retroviral antigen‐specific CD8 T cell staining

An in‐house H‐2K^b murine leukemia virus (MuLV) p15E tetramer was prepared using published sequences (MBL Life Science). For staining, 1 × 10^7^ cells were incubated with 0.04 mg/mL tetramer and 0.025 mg/mL streptavidin‐PE in 100 µL FACS buffer on ice, in the dark, for ≥30 min. After live/dead and Fc block, cells were stained for 1 h with the MuLV‐PE complex and T cell markers. CD8 T cells were identified via CD4‐negative gating to avoid interference from anti‐CD8 antibodies (clone 53–6.7) with MHC‐I tetramers.

### Human AML samples

Human BM aspirate samples were obtained from AML patients undergoing treatment with HMA and/or venetoclax (VEN) at designated time‐points (Supporting Information S2: Table 1). Written informed consent (Declaration of Helsinki) was obtained from all patients according to approved ethics from the Technische Universität München (TUM 339/21). Bone marrow mononuclear cells (BMMCs) were isolated by Ficoll density gradient centrifugation (Biocoll Separating Solution density 1.077 g/mL, Bio‐sell, BS.L 6115) and red blood cells (RBCs) were lysed with ammonium‐chloride‐potassium (ACK) lysis buffer. Cells were frozen and stored in liquid nitrogen until further use. Upon thawing, CD34^+^ positive isolation (Miltenyi Biotec) was performed to enrich AML blasts and non‐malignant hematopoietic stem cells from the sample before proceeding with downstream experiments.

### Statistical analyses

Data were analyzed and assessed for normality using GraphPad Prism v9. Normality was assessed with Kolmogorov–Smirnov and Shapiro–Wilk tests to determine use of parametric or non‐parametric methods. Specific tests are detailed in figure legends. All survival curves were compared using the two‐sided log‐rank (Mantel–Cox) test. Data are shown as mean ± SEM (excluding survival). P ≤ 0.05 was considered significant; exact P‐values are provided when appropriate.

All other methods are provided in the [Link], [Link].

## RESULTS

### AML‐intrinsic RNA‐sensing receptor signaling via MAVS is essential for 5‐AZA antileukemic activity

To evaluate the functional relevance of innate nucleic acid sensing pathways in mediating antileukemic activity of HMAs, we used CRISPR/Cas9‐mediated somatic mutagenesis to generate murine AML cell lines (C1498 and WEHI‐3B) deficient in the adaptor proteins STING or MAVS, which are critical in DNA (cGAS)‐ and RNA (RLH)‐sensing pathways, respectively (Supporting Information S1: Figure 1). We engrafted two genetically distinct syngeneic AML mouse models (WEHI‐3B in BALB/c and C1498 in C57Bl/6j) with either wild‐type (WT), Sting (*Sting* ^*−/−*^)‐ or Mavs (*Mavs* ^*−/−*^)‐deficient AML cells. The HMA, 5‐AZA (or DMSO vehicle control) was administered in a manner that recapitulates treatment regimens used in human patients (Figure 1A).

**Figure 1:**
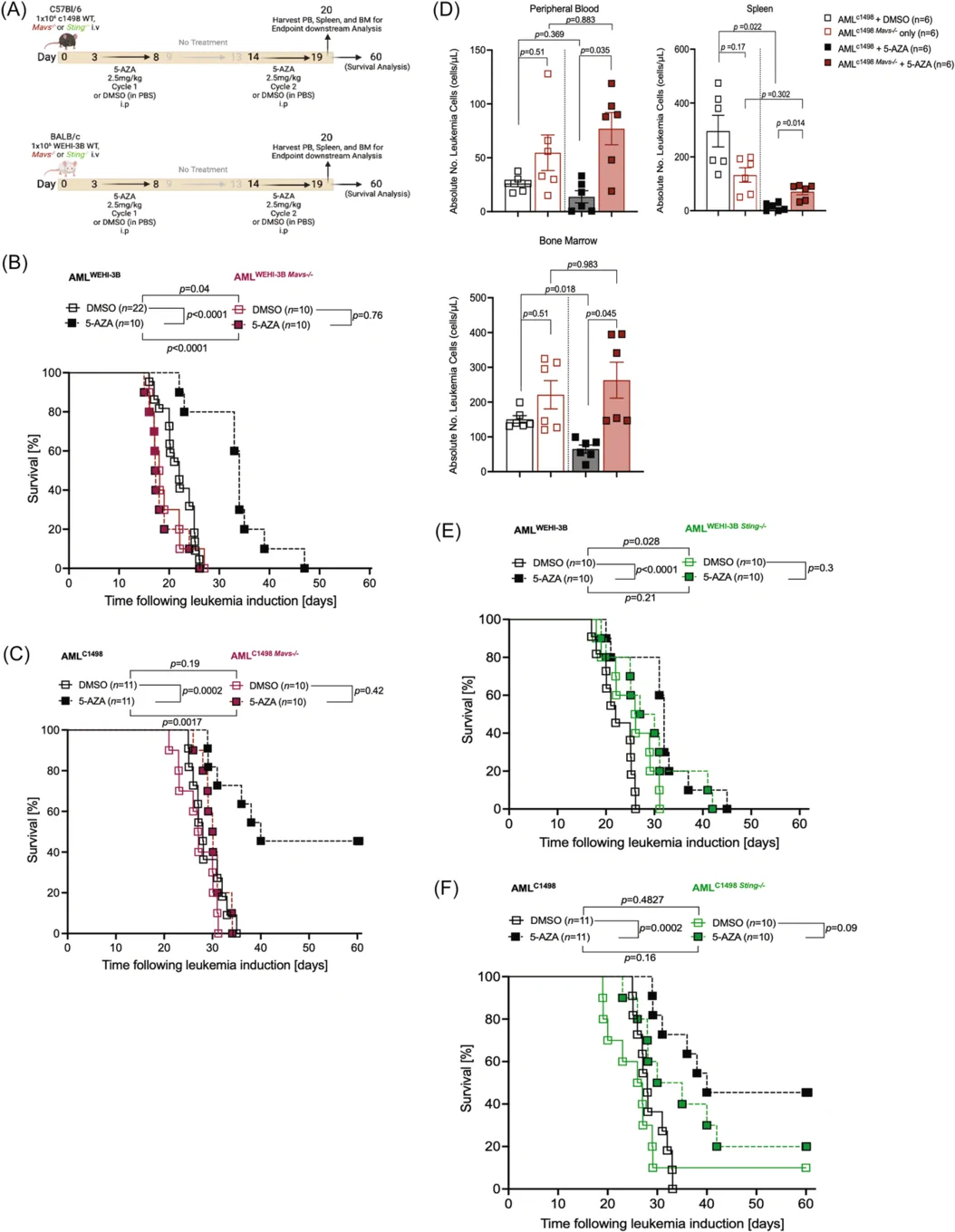
**Leukemia‐intrinsic *Mavs* deficiency abrogates the therapeutic efficacy of 5‐azacytidine (5‐AZA) in murine acute myeloid leukemia (AML) models. (A)** Experimental design: mice were intravenously (i.v) inoculated with either wild‐type (WT), *Mavs*‐ or *Sting‐*deficient leukemia cells, with WEHI‐3B cells in BALB/c mice and C1498 cells in C57BL/6j mice. Recipient animals were treated with DMSO in PBS or two cycles of 5‐AZA (2.5 mg/kg) and were monitored for longitudinal survival studies. A predefined cohort of mice was sacrificed on Day 20 to determine leukemia burden in PB, spleen, and BM by flow cytometry. **(B, C)** Kaplan–Meier survival curves for mice engrafted with either WT or *Mavs* ^*−/−*^ **(B)**, AML^WEHI‐3B^, or **(C)** AML^C1498^. Data were pooled from two independent experiments. **(D)** Quantification of leukemic cell burden in PB, spleen, and BM across different experimental groups. Statistical significance was determined using one‐way analysis of variance (ANOVA) (Kruskal–Wallis test with Dunn's multiple comparisons). **(E, F)** Kaplan–Meier curves of overall survival in mice engrafted with AML^WEHI‐3B^ **(E)** or AML^C1498^ **(F)**, comparing DMSO in PBS and 5‐AZA‐treated groups of mice bearing either WT or *Sting* ^−/−^ AML backgrounds. Data were pooled from two independent experiments. BM, bone marrow; PBS, phosphate buffered saline.

Under steady‐state conditions, in the absence of 5‐AZA treatment, mice bearing *Mavs* ^*−/−*^ leukemia exhibited a modestly more aggressive disease course, marked by reduced survival compared to those with WT leukemia. While reduction in host survival reached statistical significance (P = 0.04) only in the AML^WEHI‐3B^ model, a similar trend was observed for AML^C1498^ (Figure 1B,C). 5‐AZA treatment in WT AML^WEHI‐3B^ and AML^C1498^ bearing mice markedly prolonged survival compared to DMSO‐treated controls. Yet, 5‐AZA treatment failed to demonstrate antileukemic activity in *Mavs* ^*−/−*^ AML‐bearing mice, leading to rapid disease progression and death of the recipient animals. We directly assessed leukemic burden by flow cytometric analysis of peripheral blood (PB), spleen, and BM. Mice bearing *Mavs*‐deficient AML treated with DMSO or 5‐AZA had significantly higher leukemia cell counts compared to WT AML‐bearing mice treated with 5‐AZA (Supporting Information S1: Figure 2; Figure 1D). In contrast to the important role of AML‐intrinsic *Mavs* activity, *Sting* deficiency had only subtle effects on 5‐AZA antileukemic efficacies in both murine models (Figure 1E,F).

### Selective engagement of RIG‐I–mediated RNA‐sensing defines the molecular response of AML cells to 5‐AZA

To further elucidate the molecular mechanisms driving the differential responses to 5‐AZA treatment, we examined the expression profiles of key components of the nucleic acid sensing pathways within murine AML cells. To distinguish species‐specific effects and facilitate the translational interpretation of our findings, we also included a human AML cell line (MOLM‐13) into our analyses. While 5‐AZA elicited modest increases in transcriptional and protein levels of STING and MDA5 expression, RIG‐I/MAVS showed the most consistent upregulation both in transcriptional activity as well as protein levels across various murine and human AML cell lines (Figure 2A,B; Supporting Information S1: Figure 3). Furthermore, 5‐AZA exposure led to enhanced phosphorylation of key factors in the nucleic acid sensing pathways including IRF3, NF‐κB, and STAT1 within different AML cells. In line with activation of IRF3 and STAT1, AML cells showed potent transcriptional upregulation of IFN‐Is in response to 5‐AZA exposure *in vitro* (Figure 2C,D). 5‐AZA‐induced IFN‐I activity was completely abrogated in *Mavs*‐deficient murine AML cells (Figure 2E), supporting our above findings that RLH/MAVS‐mediated RNA‐sensing rather than the cGAS/STING DNA receptor system in AML cells is central for 5‐AZA antileukemic effects. Importantly, similar transcriptional upregulation and pathway activation of innate RNA‐sensing systems in murine and human AML cell lines was observed following decitabine (DAC) exposure, suggesting this effect is not unique to 5‐AZA but may reflect a class‐wide property of HMAs (Supporting Information S1: Figure 4). To determine whether RIG‐I/MAVS dependence reflects a broader response pattern also to genotoxic stress, we assessed WT and MAVS‐deficient AML cells exposed to clinically relevant DNA damaging chemotherapeutic agents. In contrast to HMA treatment, cytotoxic cell death occurred independently of AML‐intrinsic RIG‐I/MAVS (Supporting Information S1: Figure 5), indicating that RLH‐mediated sensing is not a universal response to DNA damage.

**Figure 2:**
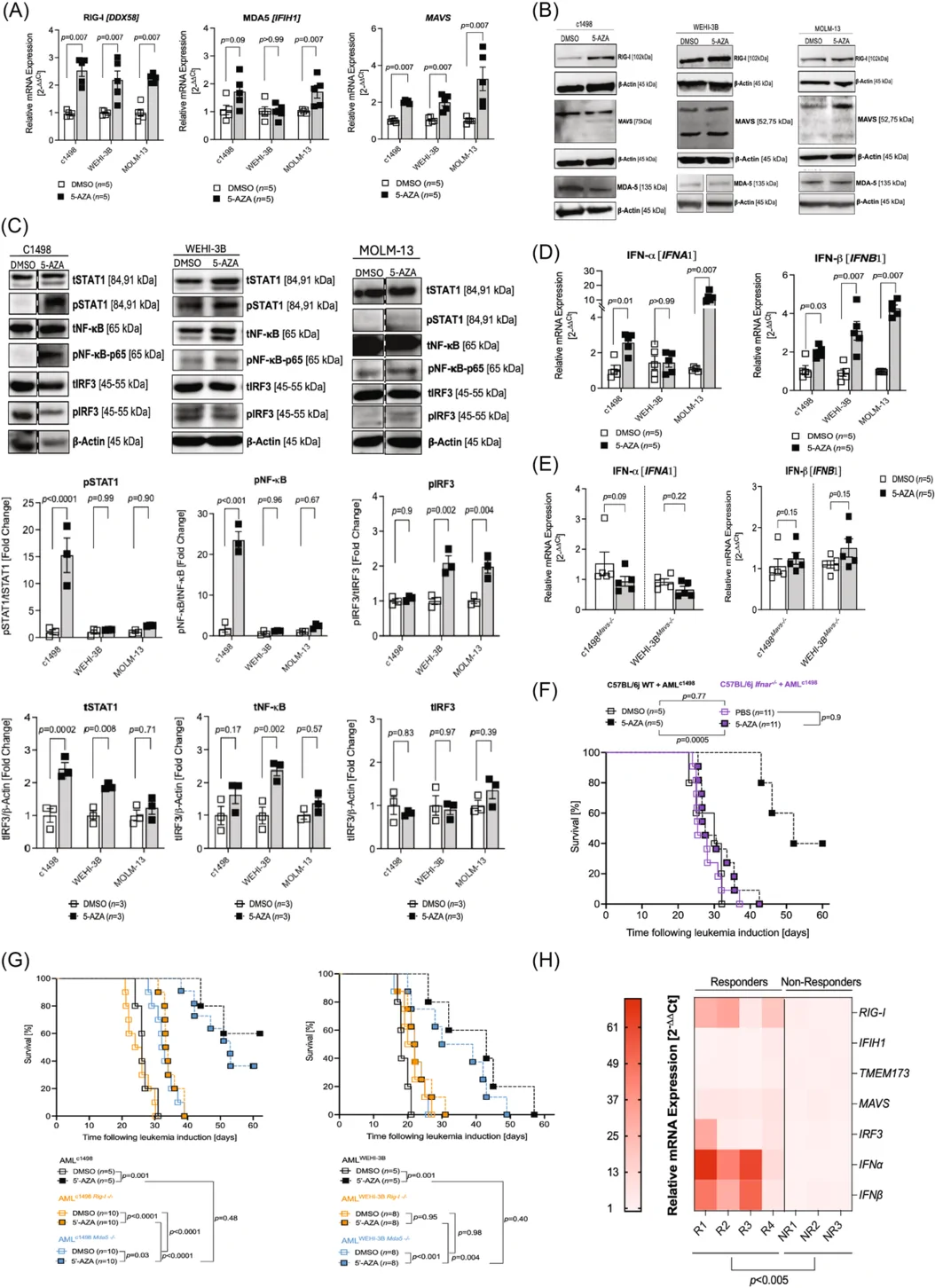
**Acute myeloid leukemia (AML) cell‐intrinsic retinoic acid‐inducible gene I (RIG‐I)/mitochondrial antiviral‐signaling protein (MAVS) but not MDA5 activity and subsequent type I interferon (IFN‐I) signaling in host cells are critical for antileukemic effects of 5‐azacytidine (5‐AZA). (A–D)** Murine and human AML cell lines (C1498, WEHI‐3B, and MOLM‐13) were treated with DMSO or 5‐AZA *in vitro*. (A) qPCR analysis of RIG‐I (DDX58), MDA‐5 (IFIH1), and MAVS expression. **(B, C)** Representative western blots showing RIG‐I‐like receptor (RLR) pathway proteins in AML cell lines and corresponding densitometric quantifications **(C**; bottom) of total and phosphorylated STAT1, NFκB‐p65, and IRF3 in C1498, WEHI‐3B, and MOLM‐13 AML cell lines treated with DMSO or 5‐AZA. Densitometric data points represent individual replicates (*n* = 3). **(D)** qPCR analysis of IFN‐α and IFN‐β in wild‐type (WT) or **(E)** *Mavs* ^*−/−*^ AML^C1498^ or AML^WEHI‐3B^ cells treated with DMSO or 5‐AZA. **(F)** Kaplan–Meier survival curves of WT and *Ifnar* ^−/−^ C57BL/6j mice inoculated with AML^C1498^ cells and treated with 5‐AZA or DMSO in phosphate buffered saline (PBS). **(G)** Kaplan–Meier curves of C57BL/6j and BALB/c mice inoculated with AML^C1498^ or AML^WEH‐3B^ WT, *Rig‐I* ^−/−^ or *Mda5* ^−/−^ cells, respectively, and treated with DMSO in PBS or 5‐AZA. Data were pooled from two independent experiments. **(H)** qPCR analysis of RIG‐I–like helicase (RLH) pathway components and IFN‐I mRNA expression in bone marrow (BM) aspirate samples from AML patients receiving hypomethylating agent (HMA) therapy with either 5‐AZA alone or VEN/5‐AZA. CD34^+^ cells (including AML blasts and non‐malignant hematopoietic stem cells) were isolated from matched patient samples obtained at diagnosis (pre‐treatment) and following HMA treatment. “Relative mRNA expression” refers to expression in on‐treatment samples normalized to expression levels in the corresponding diagnostic (baseline) sample from the same patient. Data are stratified by clinical response status (responder vs. non‐responder) to therapy. **(A, D** and **E)** P‐values were calculated using a two‐way analysis of variance (ANOVA) followed by Sidak's multiple comparisons test. **(C)** P‐values were determined using one‐way ANOVA (Kruskal–Wallis test with Dunn's multiple comparisons). For **(H)** Benjamini–Hochberg false discovery rate (FDR) correction was additionally applied.

In line with previous reports of autocrine feedback loops and upregulation of certain IFN‐stimulated central components of the RIG‐I pathway,^22^ we found that intrinsic *Mavs* activity (as a prerequisite for the downstream IFN‐I response) was indeed critical for the enhanced transcriptional activity of both RIG‐I and MDA5 upon 5‐AZA exposure in AML cells (Supporting Information S1: Figure 6). Yet, tumor‐intrinsic Type I IFN production can act both in an autocrine and paracrine fashion, potentially influencing bystander host immune cells.^23^, ^24^ Indeed, the survival benefit typically observed upon 5‐AZA treatment was completely abrogated in AML‐bearing mice with host deficiency for the common interferon α/β receptor subunit 1 (*Ifnar1* ^*−/−*^) (Figure 2F). These data demonstrate that functional IFN‐I signaling in host cells is critical for 5‐AZA‐mediated leukemia control.

Different members of the RLH RNA receptor family can bind distinct RNA types and structures. To identify the RNA receptor primarily responsible for mediating the antileukemic effects of 5‐AZA, we inoculated mice with *Rig‐I* or *Mda5‐*deficient AML cells. Notably, 5‐AZA treatment‐associated survival benefits were strongly reduced in mice bearing *Rig‐I* ^*−/−*^ but not *Mda5* ^*−/−*^ AML (Figure 2G). Taken together, these findings indicate that AML cell‐intrinsic RIG‐I/MAVS activity and subsequent IFN‐I signaling in host cells are critical for the antileukemic effects of 5‐AZA. Consistent with these findings, in a small case series of AML patients undergoing 5‐AZA monotherapy or combination treatment with the BCL‐2 inhibitor VEN, retrospective analysis of CD34^+^ isolated pre‐ and on‐treatment BM samples demonstrated increased transcriptional activity of RIG‐I and downstream IFN‐I signaling pathways in treatment responders compared to non‐responding AML patients (Figure 2H and Supporting Information S2: Table 1).

### 5‐AZA enhances expression of ERV RNA transcripts that directly interact with the RIG‐I receptor in AML cells

In solid tumors, 5‐AZA treatment has been shown to induce expression of ERV transcripts, specifically in melanoma, choriocarcinoma, and endometrial cancer cells.^9^, ^10^ In a targeted screen of known ERVs in human and murine AML cell lines, we here found that 5‐AZA exposure resulted in similar patterns of enhanced transcriptional activity of endogenous retroviral elements in AML (Figure 3A). ERV expression was likewise elevated following DAC exposure, reinforcing the idea that HMA‐driven epigenetic reprogramming, rather than compound‐specific activity, underlies this response (Supporting Information S1: Figure 7A). This phenomenon seemed largely exclusive to malignantly transformed leukemic cells—potentially due to their increased genetic instability—as 5‐AZA treatment of steady‐state mice did not trigger significant ERV mRNA upregulation (Supporting Information S1: Figure 7B,C). While we observed minor trends toward increased expression in selected ERVs in both mouse strains, these changes were not statistically significant. Consistently, 5‐AZA treatment did not result in IFN‐I activation or transcriptional priming of RLH pathways in non‐transformed hematopoietic cells (Supporting Information S1: Figure 7D,E).

**Figure 3:**
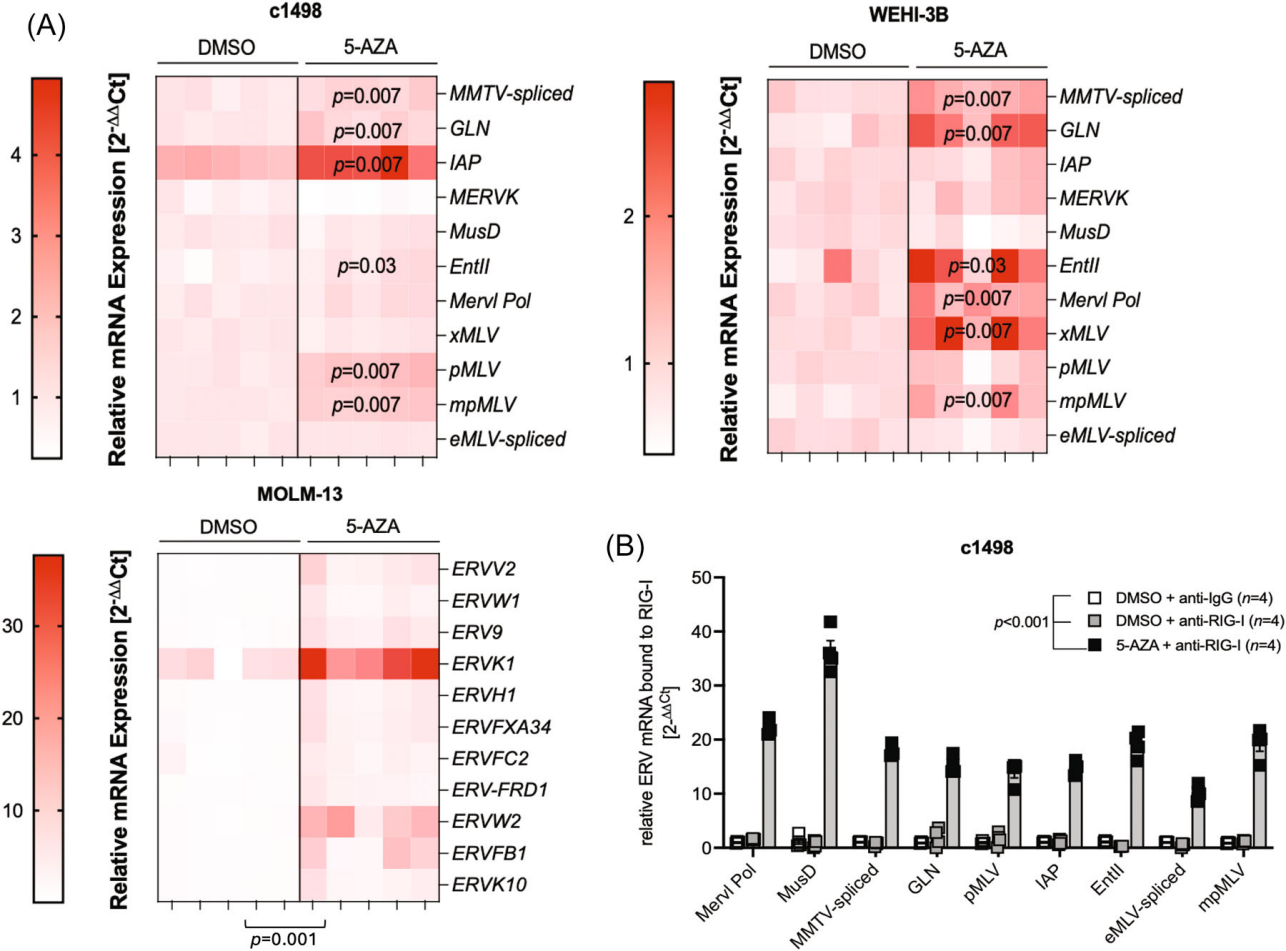
**5‐Azacytidine (5‐AZA) enhances expression of endogenous retroviral (ERV) mRNA transcripts that directly interact with the retinoic acid‐inducible gene I (RIG‐I) receptor in acute myeloid leukemia (AML) cells. (A)** qPCR analysis of well‐characterized murine and human ERV transcripts in AML^C1498^, AML^WEHI‐3B^, and AML^MOLM‐13^ cells treated with DMSO or 5‐AZA. **(B)** qPCR analysis of RIG‐I–bound murine ERV transcripts in AML^C1498^ cells treated with DMSO or 5‐AZA. (A) P‐values were calculated using a two‐sided unpaired non‐parametric Student's *t*‐test (Mann–Whitney) or parametric Student's *t*‐test, and **(B)** P‐values were determined using one‐way analysis of variance (ANOVA) (Kruskal–Wallis test with Dunn's multiple comparisons).

To explore whether 5‐AZA‐increased abundance of ERV transcripts could directly trigger RIG‐I pathway activation, we performed RNA‐binding protein (RBP) immunoprecipitation from 5‐AZA exposed AML^C1498^ cell lysates. Immunoprecipitation with a bead‐coupled anti‐RIG‐I monoclonal antibody resulted in the specific enrichment of RIG‐I protein; polyclonal anti‐mouse IgG was used as a negative control for unspecific protein recovery (Supporting Information S1: Figure 8). Co‐isolated RBP‐associated RNA molecules were extracted from the protein precipitate and identified by qPCR analysis. Here, we found strong enrichment of ERV RNA transcripts in the RIG‐I–associated RNA interactome from 5‐AZA‐exposed AML cells (Figure 3B). These findings suggest that ERV RNA transcripts upregulated by 5‐AZA‐induced epigenetic regulation may directly bind to and potentially activate the RIG‐I receptor in AML cells.

### 5‐AZA‐induced innate and adaptive immune surveillance relies on AML‐intrinsic MAVS activity

To investigate how RLH/MAVS‐dependent signaling shapes the immune system in response to 5‐AZA, we performed high‐dimensional mass cytometry profiling of BM immune cell populations upon HMA treatment in WT and *Mavs* ^*−/−*^ AML^C1498^‐bearing mice. UMAP projection showed that 5‐AZA treatment induced broad remodeling of the WT immune landscape compared with DMSO‐treated controls. In contrast, immune compartmentalization in *Mavs* ^*−/−*^ AML‐bearing mice remained comparatively diffuse, indicating that AML‐intrinsic MAVS is required for the immune reorganization elicited by HMA therapy (Figure 4A). Quantitative analysis of cluster frequencies demonstrated that in the absence of 5‐AZA treatment, WT and *Mavs* ^*−/−*^ AML mice showed largely similar immune compositions in the BM, suggesting that MAVS deficiency alone does not overtly reshape basal immune homeostasis in this model. However, in the context of 5‐AZA treatment, accumulation of several lymphoid subsets including CD8^+^ T cells, effector memory CD8^+^ T cells, γδ T cells, activated NK cells, and B cells was abrogated in animals bearing *Mavs* ^*−/−*^ AML (Figure 4B). Also, these showed increased abundance of inflammatory monocytes in the BM. Furthermore, 5‐AZA treatment resulted in a reduction of macrophages; however, this was not significantly dependent on AML cell‐intrinsic MAVS activity. In summary, the observed broad changes in the immune cell composition of the BM in AML‐bearing mice suggest that AML cell‐intrinsic MAVS is necessary for coordinated immune reprogramming in response to 5‐AZA.

**Figure 4:**
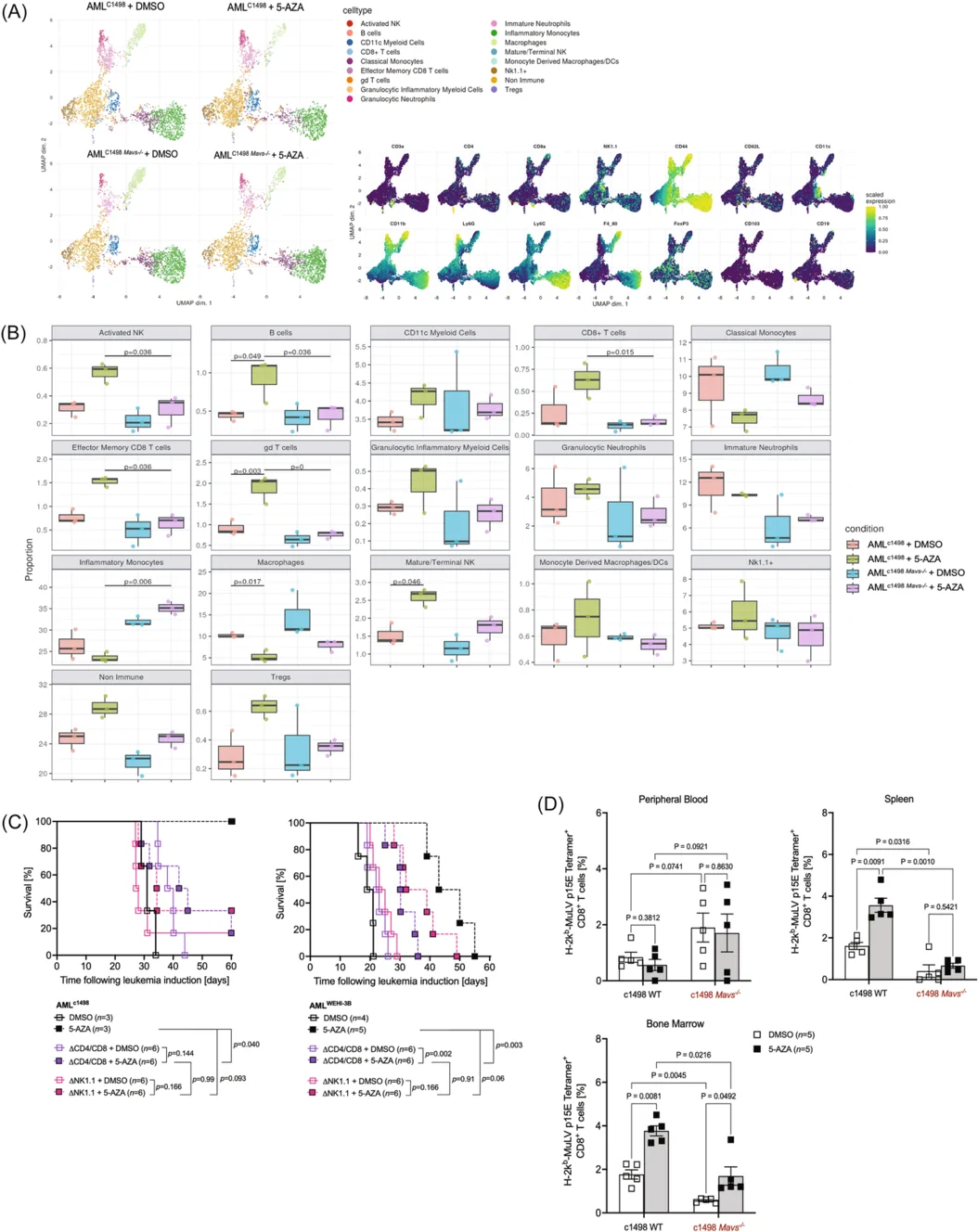
**5‐Azacytidine (5‐AZA)‐induced innate and adaptive immune surveillance relies on acute myeloid leukemia (AML)‐intrinsic mitochondrial antiviral‐signaling protein (MAVS) activity. (A, B)** Wild‐type (WT) or *Mavs* ^*−/−*^ AML^C1498^ bearing mice were treated with 5‐AZA, and immune cell subpopulations in the bone marrow (BM) were analyzed by mass cytometry (CyTOF). (A) UMAP visualization (left panel) depicting the projections of immune cell clusters from the BM based on treatment and genotype. Feature plots (bottom right panel) show the expression level of different marker genes relevant for the characterization of the immune clusters. **(B)** Box‐and‐whisker plots depicting the relative frequency (% proportions of total CD45^+^) of each identified cluster from each genotype and treatment condition. P‐values were determined using one‐way analysis of variance (ANOVA) (Kruskal–Wallis test with Dunn's multiple comparisons). **(C)** Kaplan–Meier curves comparing survival of mice bearing WT AML^C1498^ or AML^WEHI‐3B^ cells treated with DMSO in phosphate buffered saline (PBS) or 5‐AZA (2.5 mg/kg) under conditions of an intact immune system, CD4/CD8 T‐cell depletion, or NK‐cell depletion. Data represent two independent experiments. **(D)** Expansion of H‐2k^b^ MuLV ERV p15E MHC multimer‐specific CD8^+^ T‐cell responses in peripheral blood (PB), spleen, and BM of WT or *Mavs* ^*−/−*^ AML^C1498^ bearing mice upon treatment with DMSO or 5‐AZA were determined by flow cytometry. Data were pooled from two independent experiments. P‐values were calculated using a two‐way ANOVA (Sidak's multiple comparisons test).

While several lymphoid and myeloid populations showed treatment‐dependent shifts, cytotoxic T and NK cells were amongst the most consistently and prominently expanded effector subsets in the BM upon 5‐AZA treatment. Given the pre‐existing mechanistic and clinical evidence^25^, ^26^, ^27^ suggesting T‐ and NK‐cell activation as important immune effectors of HMA responses, we focused functional validation in the context of our AML models on these two cell subtypes. In line with the above findings, antibody‐mediated depletion of either T‐ or NK cells in AML‐bearing mice largely abrogated the 5‐AZA treatment‐associated survival benefit, demonstrating that antileukemic activity of 5‐AZA indeed relies on T‐ and NK‐cell–mediated immunosurveillance (Figure 4C). Multi‐parameter flow cytometry analyses confirmed increased abundance and enhanced activation levels of both CD4^+^ and CD8^+^ T cells as well as NK cells in the spleen and BM of AML‐bearing mice following 5‐AZA treatment (Supporting Information S1: Figure 9).

Previous studies suggested that gene products derived from ERVs may form a reservoir of T‐cell targets in hematological malignancies including AML.^13^ A peptide derived from the envelope protein (p15E) of an endogenous ecotropic MuLV has previously been identified as an immunodominant epitope of MuLV‐induced lymphomas. Here, we found that 5‐AZA treatment of AML‐bearing mice led to a robust expansion of H‐2K^b^ MuLV ERV p15E‐specific CD8^+^ T cells (Figure 4D). Accumulation of 5‐AZA‐induced ERV peptide‐reactive T cells at anatomical sites of high leukemic burden (BM and spleen) was markedly reduced in animals bearing *Mavs* ^*−/−*^ AML compared to WT. Collectively, these findings establish RLH/MAVS signaling as a pivotal mediator of 5‐AZA‐induced leukemia immune surveillance, facilitating both innate and adaptive immune activation to suppress AML progression.

### 5‐AZA‐resistance is associated with ERV repression and blunted nucleic acid receptor signaling in AML cells

Secondary resistance to HMAs is a common event in the treatment of patients with AML. To investigate whether such acquired resistance to 5‐AZA (5‐AZA‐R) coincides with defects in nucleic acid sensing and ERV activation, we established AML cell lines resistant to 5‐AZA. Western blot analysis of AML^C1498^ and AML^WEHI‐3B^ cells confirmed that in 5‐AZA‐R cells, 5‐AZA‐induced suppression of the DNA methyltransferase DNMT1 was lost, which is a hallmark of epigenetic resistance (Figure 5A). BALB/c mice inoculated with 5‐AZA‐R AML^WEHI‐3B^ demonstrated no therapeutic response or survival benefit from 5‐AZA treatment, in contrast to those inoculated with WT AML^WEHI‐3B^ leukemia, indicating a resistant phenotype (Supporting Information S1: Figure 10). Notably, qPCR analysis revealed that ERV transcript levels, which were robustly induced by 5‐AZA in steady‐state AML cells, were significantly repressed in murine and human 5‐AZA‐R AML (Figure 5B). In AML patients, we found enhanced expression of ERV transcripts in BM samples from responders to HMA treatment compared to non‐responders, as suggested previously^28^ (Figure 5C). Analysis of a publicly available data set of global methylation signatures in patients with high‐risk MDS or secondary AML undergoing HMA treatment^29^ showed marked locus demethylation across the ERV1, ERVK, and ERVL families in clinical responders (Supporting Information S2: Table 7; Supporting Information S1: Figure 11; Figure 5D). In contrast, non‐responder patients failed to undergo epigenetic remodeling, maintaining high relative methylation profiles. This clinical failure to demethylate retroviral elements closely phenocopies the hypermethylator, ERV‐repressed state observed in 5‐AZA‐R cell lines. In these models, loss of ERV induction correlated with impaired nucleic acid receptor signaling. RIG‐I and MAVS expression were markedly downregulated in 5‐AZA‐R AML cells (Figure 5E). Western blot analysis demonstrated that the receptors as well as critical components of both the RIG‐I‐like receptor (RLR) and cGAS‐STING pathways were significantly downregulated in 5‐AZA‐R AML, suggesting that acquired 5‐AZA‐R is associated with a broad suppression pattern of leukemia‐intrinsic nucleic acid sensing (Figure 5F). Ultimately, low phosphorylation of central downstream transcription factors resulted in a profound reduction of IFN‐I transcriptional activity in 5‐AZA‐R AML cells (Figure 5G). In summary, these data reveal a potential immune evasion mechanism in 5‐AZA‐R AML, whereby lack of ERV upregulation as well as subsequently blunted nucleic acid receptor signaling contribute to therapeutic failure.

**Figure 5:**
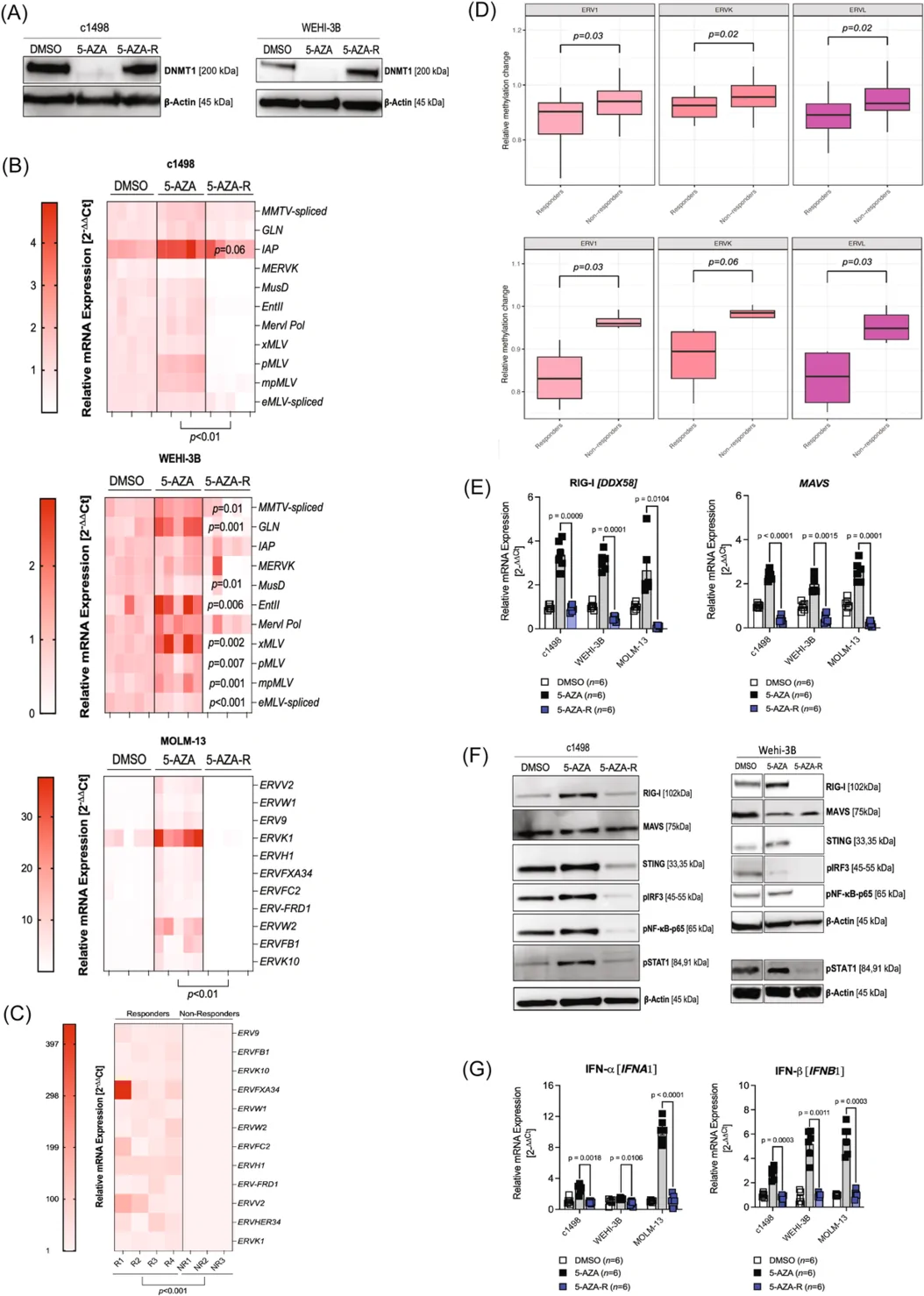
**5‐Azacytidine (5‐AZA) resistance is associated with endogenous retroviral (ERV) repression and blunted nucleic acid receptor signaling in acute myeloid leukemia (AML) cells.** For all conditions pertaining to resistance to 5‐AZA (5‐AZA‐R), cells were treated with 5‐AZA once a week during standard tissue culture to maintain the resistant line; however, no further 5‐AZA treatment was administered during the experimental setup. **(A)** Representative western blots showing DNMT1 protein levels in native DMSO and 5‐AZA‐treated or 5‐AZA‐R AML^C1498^ and AML^WEHI‐3B^ cells. qPCR analysis of **(B)** murine and human ERV transcripts and **(C)** in on‐treatment bone marrow biopsy samples from CD34^+^ blasts from AML patients undergoing hypomethylating agent (HMA) treatment. Data are presented by leukemia response status to HMA treatment. **(D)** Boxplots represent the relative DNA methylation changes for ERV1, ERVK, and ERVL retroviral element families compared between clinical responders and non‐responders. Relative changes were determined against the paired diagnosis sample for each patient. The top panel represents all selected high‐risk myelodysplastic syndrome (MDS) and secondary AML samples. The bottom panel depicts only the secondary AML samples. **(E)** Retinoic acid‐inducible gene I (RIG‐I) and mitochondrial antiviral‐signaling protein (MAVS) expression in wild‐type (WT) AML^C1498^, AML^WEHI‐3B,^ and AML^MOLM‐13^ cells treated with DMSO or 5‐AZA, compared to their 5‐AZA‐R counterparts. **(F)** Representative western blots showing RIG‐I‐like receptor (RLR) and cyclic GMP‐AMP synthase (cGAS)‐stimulator of IFN genes (STING) pathway protein levels in AML^C1498^ and AML^WEHI‐3B^ cell lines treated with DMSO, or 5‐AZA, compared to 5‐AZA‐R cells. **(G)** qPCR analysis of interferon (IFN)‐α and IFN‐β expression in the AML cell lines under the same conditions as **(E)**. **(B, C, D)** P‐values were calculated using a non‐parametric Wilcoxon test. **(E, G)** Data were pooled from two independent experiments. P‐values were calculated using a two‐sided unpaired non‐parametric Student's *t*‐test (Mann–Whitney) or parametric Student's *t*‐test.

### ATRA treatment can re‐sensitize RIG‐I/MAVS signaling in 5‐AZA‐resistant AML

RIG‐I was initially identified in a screen of genes with potent transcriptional upregulation in acute promyelocytic leukemia (APL) cells in response to the eponymous vitamin A metabolite all*‐trans‐*retinoic acid (ATRA).^30^, ^31^ To test whether pharmacologic reactivation of the RIG‐I pathway might restore leukemia susceptibility, we exposed 5‐AZA‐R AML cells to ATRA. Indeed, ATRA treatment resulted in robust upregulation of RIG‐I expression, with particularly prominent effects in 5‐AZA‐R AML cells (Figure 6A,B). In line with proapoptotic signaling of RLHs, pronounced ATRA‐induced RIG‐I upregulation enhanced apoptotic cell death in 5‐AZA‐R AML cells (Figure 6C).

**Figure 6:**
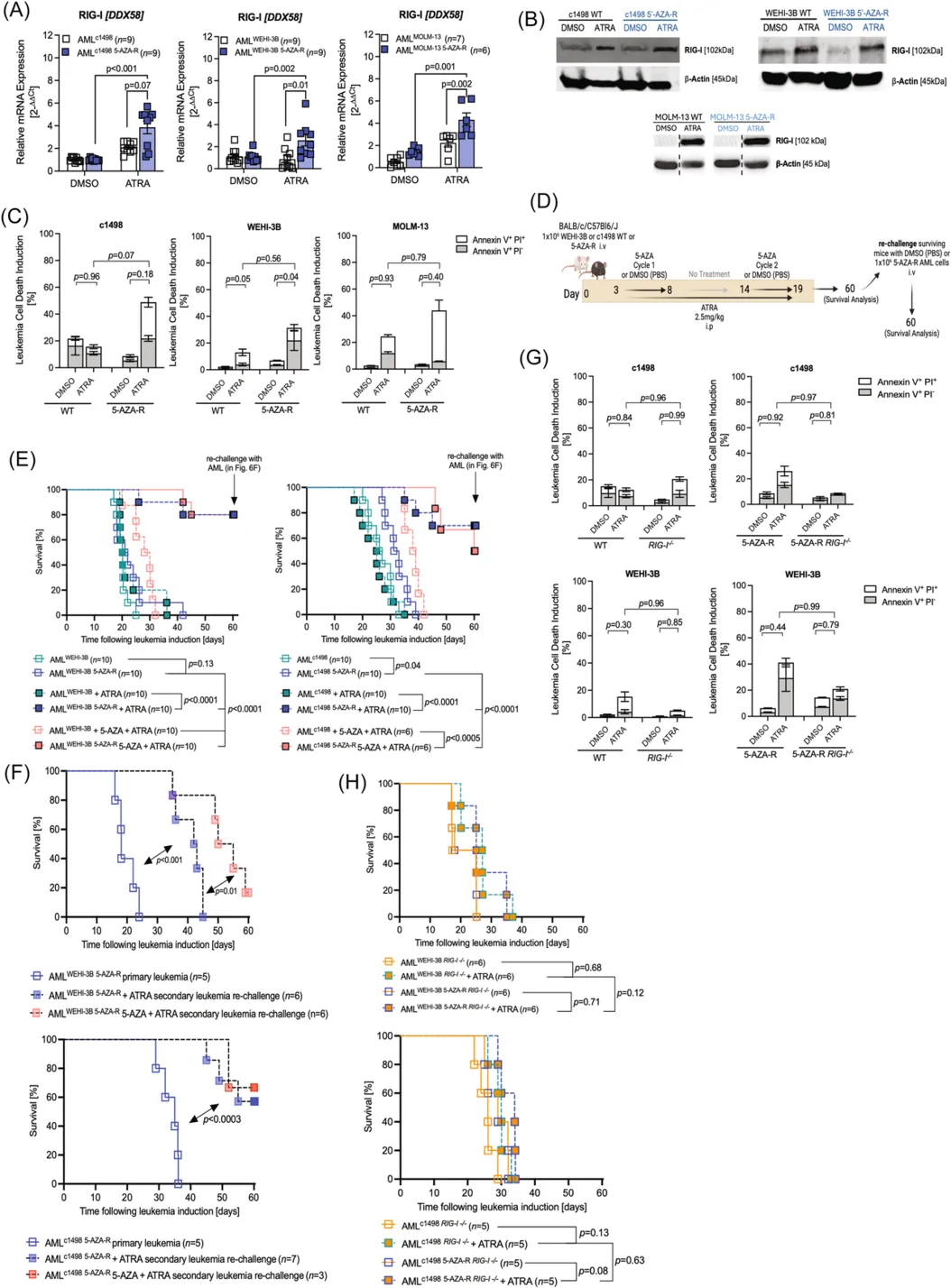
**All*‐trans* retinoic acid (ATRA) modulates retinoic acid‐inducible gene I (RIG‐I)/mitochondrial antiviral‐signaling protein (MAVS) signaling in acute myeloid leukemia (AML) cells with acquired resistance to 5‐azacytidine (5‐AZA‐R). (A)** qPCR analysis of RIG‐I expression in wild‐type (WT) and 5‐AZA‐R AML^C1498^, AML^WEHI‐3B^, and AML^MOLM‐13^ cells treated with DMSO or 100 nM ATRA for 24 h. Data represent three independent experiments; P‐values were calculated using a two‐sided Mann–Whitney test. **(B)** Representative western blots showing RIG‐I protein levels in AML^C1498^ and AML^WEHI‐3B^ cell lines treated with DMSO or 100 nM ATRA for 24 h. **(C)** Stacked bar graphs showing Annexin V/PI staining in WT and 5‐AZA‐R AML^C1498^, AML^WEHI‐3B^, and AML^MOLM‐13^ cells after 24 h of DMSO or 100 nM ATRA. **(D)** Schematic overview of the *in vivo* AML induction model in BALB/c mice using AML^WEHI‐3B^ or AML^C1498^ WT or 5‐AZA‐R AML cells. Mice were treated daily with ATRA (2.5 mg/kg) or DMSO in phosphate buffered saline (PBS) from Days 3 to 19, followed by survival monitoring through Day 60. Surviving mice were then re‐challenged with 1 × 10^6^ AML^WEHI‐3B^ AML^C1498^ cells or PBS and monitored for an additional 60 days. **(E)** Kaplan–Meier curves for mice engrafted with AML^WEHI‐3B^ (left) or AML^C1498^ (right) WT or 5‐AZA‐R cells treated with ATRA, DMSO in PBS, or 5‐AZA + ATRA. **(F)** Kaplan–Meier curves from re‐challenge experiments (top; AML^WEHI‐3B^ and bottom; AML^C1498^). Of the 5‐AZA‐R AML mice treated with ATRA in **(E)**, the surviving mice from the primary challenge were re‐challenged with 1 × 10^6^ AML^WEHI‐3B or^ AML^C1498^ 5‐AZA‐R cells. Similarly, the surviving 5‐AZA‐R AML mice at Day 60 that were treated with 5‐AZA + ATRA **(E)** were re‐challenged with 1 × 10^6^ AML^WEHI‐3B^ or AML^C1498^ 5‐AZA‐R AML. Five mice were inoculated with primary AML^WEHI‐3B^ or AML^C1498^ 5‐AZA‐R cells for the first time as experimental controls. All mice were monitored for up to 60 days. **(G)** Stacked bar graph depicting Annexin V/PI expression in WT, 5‐AZA‐R, and *Rig‐I*–deficient WT and 5‐AZA‐R AML^C1498^ and AML^WEHI‐3B^ cells treated with DMSO or 100 nM ATRA for 24 h. **(H)** Kaplan–Meier curves for mice engrafted with WT or 5‐AZA‐R *Rig‐I*–deficient AML^WEHI‐3B^ (top) or AML^C1498^ (bottom) cells and treated with DMSO in PBS or ATRA. **(A, C, D)** P‐values were calculated using a two‐sided unpaired non‐parametric Student's *t*‐test (Mann–Whitney) or parametric Student's *t*‐test. **(E, F, H)** Data were pooled from two independent experiments.

To assess the *in vivo* therapeutic implications of RIG‐I reactivation, mice were inoculated with 5‐AZA‐R AML cells and subsequently subjected to ATRA treatment or its combination with 5‐AZA (Figure 6D). Remarkably, ATRA monotherapy or its combination with 5‐AZA significantly prolonged survival in 5‐AZA‐R AML‐bearing mice, whereas WT AML‐bearing mice exhibited no therapeutic benefit from either treatment (Figure 6E). To assess long‐term effects, mice that survived the primary 5‐AZA‐R AML challenge following fixed duration ATRA or ATRA/5‐AZA treatment were re‐inoculated with 5‐AZA‐R AML. In the absence of additional therapy, these previously treated survivors exhibited improved leukemia control compared to naïve mice inoculated with 5‐AZA‐R AML for the first time (primary challenge) (Figure 6F). Initial treatment with the combination of 5‐AZA and ATRA was associated with a statistically non‐significant trend to further improve host survival upon re‐challenge with 5‐AZA‐R AML cells. Collectively, these data suggest that AML cells with acquired AZA resistance are particularly susceptible to ATRA‐induced proapoptotic priming and antileukemic activity.

To definitively establish that the pronounced vulnerability of 5‐AZA‐R AML toward ATRA is contingent upon RIG‐I reactivation, we studied the impact of ATRA on 5‐AZA‐R AML cells with irreversible genetic deficiency for RIG‐I. Indeed, ATRA treatment failed to induce apoptosis in RIG‐I–deficient 5‐AZA‐R AML^C1498^ or AML^WEHI‐3B^ cells (Figure 6G). Consistently, the previously observed leukemia control and associated host survival benefit conferred by ATRA treatment was entirely abolished in mice bearing RIG‐I–deficient 5‐AZA‐R AML (Figure 6H). Altogether, these data provide evidence that ATRA can modulate the RIG‐I/MAVS pathway and restore proapoptotic signaling in AML cells with acquired 5‐AZA‐R. These findings underscore the therapeutic potential of targeting nucleic acid sensing pathways via alternate agents to circumvent epigenetic therapy resistance in AML.

## DISCUSSION

In this work, we provide mechanistic evidence that aberrant RNA expression and subsequent RIG‐I–mediated IFN‐I release by AML cells are indeed critical for the development of antileukemic T‐cell–driven immunity and subsequent AML control upon HMA treatment. This aligns with previous studies linking RLH RNA‐sensing systems to antileukemic activity. *In vitro* transcribed 5′‐triphosphate RNA targeting RIG‐I alone induced potent T‐cell–mediated leukemia control in mice.^32^ However, this study focused on systemic RIG‐I stimulation in host immune cells, leaving the role of AML‐intrinsic activity unclear. In a retrospective analysis of two heterogeneous cohorts of patients with chronic or AML or MDS, responders to HMA treatment showed transcriptional upregulation of certain transposable elements including endogenous retroviral long terminal repeats (LTRs) and a higher IFN‐I signature in enriched malignant cells.^28^ Furthermore, *in vitro* transcribed RNA of selected LTRs induced strong expression of ISGs including RIG‐I and MDA5 in cancer cell lines.^28^ Unlike previous studies in solid tumors implicating MDA5 in sensing HMA‐induced ERV RNA,^9^, ^10^ our AML data indicate that HMA‐induced ERV‐derived RNAs potently engage RIG‐I. This apparent discrepancy may arise from varying pathway expression and activity in different cancer entities, as well as differences in aberrantly expressed RNAs (e.g., length and modifications) that may dictate which RLH is primarily activated.

Potent pathway activation with synthetic STING agonists can directly induce AML cell apoptosis and T‐cell immunity in mice^33^, ^34^ and improve immunotherapy with T‐cell–recruiting bispecific antibodies.^35^ Herein, AML cells were not a priori fully responsive to STING activation, but T‐cell cytokines sensitized them toward full‐scale IFN‐I induction downstream of STING. However, we found that the cGAS/STING pathway contributed minimally to HMA‐induced IFN‐I signaling and had no appreciable impact on therapeutic efficacy in AML. This aligns with a previous report that HMA treatment of solid tumor cells resulted only in little accumulation of immunostimulatory cytosolic dsDNA.^10^ Conversely, others suggested that 5‐AZA treatment in AML can lead to cytoplasmic relocation of transposon DNA and STING‐dependent T‐cell cytotoxicity.^36^ As proapoptotic functions of intrinsic STING are determined by signaling strength,^37^ it remains unclear to which extent HMA treatment of AML can induce sufficient cytosolic ERV DNA intermediates to relevantly prime the cGAS/STING pathway. As the activity of STING is tightly regulated beyond its expression level, post‐translational modifications may blunt STING function in a cell‐specific manner.^38^, ^39^

We identified downregulation of RIG‐I pathway components and blunted IFN‐I response in AML cells as a mechanism for acquired 5‐AZA‐R. Consistently, a recent study demonstrated gradual *RIG‐I* promoter hypermethylation in human AML cell lines under DAC pressure, suggesting a stepwise selection of cells with defective RIG‐I/IFN‐I signaling.^40^ Furthermore, we demonstrated that 5‐AZA–resistant AML cells developed particular vulnerability to ATRA, which induced potent AML cell death through RIG‐I pathway reactivation. Retinoids have been reported to induce RIG‐I transcription via RARE motifs in its promoter,^31^, ^41^ and our data provide functional validation in the context of HMA resistance. This aligns with the above‐mentioned report where a high‐throughput screen identified ATRA to selectively kill DAC‐resistant AML cells but not the parental (DAC‐sensitive) cell line.^40^ Interestingly, this work suggested that global DNA hypomethylation was maintained in AML cells beyond acquired DAC resistance, reflecting some sort of long‐term epigenetic memory. This was associated with sustained abundance of selected (ERV‐derived) dsRNA in DAC‐resistant AML cells which—upon ATRA‐mediated priming of the RIG‐I pathway—induced IFN‐I–dependent leukemia cell death. Another study suggested synergistic induction of transposable elements including ERVs in AML cell lines by DAC in combination with ATRA.^42^ These findings contrast our data in which a targeted ERV screen showed blunted rather than accumulated ERV RNA expression in 5‐AZA–resistant AML cells. It currently remains unclear whether this discrepancy derives from differences between AML cell lines (with varying underlying genetic aberrations) and the used HMA therapy or whether it is reflecting a selection bias in our targeted ERV screen.

Independent of the exact dsRNA species that engage RIG‐I following ATRA‐induced reactivation in HMA‐resistant cells, combining HMAs with ATRA has shown promising clinical activity. In randomized clinical trials, addition of ATRA to DAC provided significant clinical benefit over HMA monotherapy in elderly AML patients and in MDS patients with excess blasts.^43^, ^44^ Importantly, these studies explored concurrent treatment with DAC and ATRA in treatment‐naïve patients and did not restrict ATRA treatment to patients with pre‐existing acquired HMA resistance. Our proposed mechanistic model emphasizes targeting of a distinct biological window: the immediate emergence of acquired HMA resistance, leveraging preserved epigenetic memory to trigger RIG‐I–mediated IFN‐I–dependent apoptotic AML cell death. Unlocking the full clinical potential of ATRA combination therapies may require careful patient stratification and treatment sequencing relative to prior HMA failure. Importantly, our delineation of RIG‐I/MAVS pathway attenuation as a defining feature of HMA‐refractory/relapsed AML establishes a compelling framework for patient stratification. Prospective interrogation of diagnostic specimens—via targeted transcriptomic panels or quantitative PCR assays for ERV expression and RIG‐I/MAVS signature genes—could unmask subclinical or emergent resistance, thereby informing appropriate inclusion of ATRA or RIG‐I agonists to forestall therapeutic failure.

In clinical practice, HMAs are often used in combination with the BCL‐2 inhibitor VEN in elderly AML patients as a first‐line therapy given its superior remission rates and overall survival compared to HMA monotherapy.^45^ Interestingly, agonistic RIG‐I pathway activation synergized with Bcl‐2 silencing to enhance killing of solid tumors in mice^46^ suggesting that RIG‐I activation in AML cells may act in synergy with VEN. Whether HMA‐induced activation of RIG‐I/IFN‐I signaling in AML cells contributes to the beneficial effects of VEN combination therapy warrants further investigation. Nonetheless, preliminary data from an ongoing single‐arm Phase 2 clinical trial indicate that the combination of an HMA, VEN, and ATRA yields high response rates in patients with newly diagnosed AML.^47^ Interestingly, a recent study proposed a novel indirect mechanism of ATRA via microenvironmental reprogramming,^48^ suggesting that ATRA may similarly exert dual antileukemic pressure directly re‐sensitizing blast‐intrinsic RIG‐I/MAVS immune pathways while concurrently disrupting supportive stromal niche signaling.

Our study has technical limitations. Our *in vivo* mechanistic and therapeutic evaluation relies heavily on murine AML models. Although these models are highly robust and widely accepted, they may not fully capture the profound heterogeneity and complex clonal architecture of human AML. While we validated key pathway elements using selected human AML cell lines, our access to primary human AML patient samples was limited. Given that primary patient blasts exhibit diverse baseline epigenetic configurations and variable microenvironmental dependencies, future prospective validation in larger cohorts of patient‐derived primary cells and xenograft models is required to consolidate the translational breadth of this therapeutic strategy.

Overall, our study advances the fundamental understanding of the antileukemic effects of HMA by elucidating its reliance on the innate RNA‐sensing machinery and ultimately highlights a novel mechanism of resistance linked to the suppression of this pathway. By integrating epigenetic modulation with strategies to reactivate innate immunity, our findings provide a rationale to develop combination therapies that leverage the RIG‐I/MAVS/IFN‐I axis to enhance clinical outcomes for patients with AML.

## AUTHOR CONTRIBUTIONS

**Nadia El Khawanky**: Conceptualization; investigation; funding acquisition; writing—original draft; writing—review and editing; visualization; validation; methodology; software; formal analysis; project administration; data curation; supervision; resources. **Tatiana Nedelko**: Investigation; project administration; methodology; data curation. **Giada Mandracci**: Investigation; methodology; formal analysis; data curation. **Yunus Barth**: Methodology; data curation. **Yuan Jiang**: Methodology. **Kai Wang**: Methodology. **Marc Rosenbaum**: Methodology; formal analysis; software; data curation; resources. **Christin Weiß**: Methodology; software; formal analysis; data curation. **Christoph Geisenberger**: Software; methodology; formal analysis. **Stefan Enßle**: Methodology. **Ana M. Gómez Martínez**: Methodology; software; formal analysis. **Sascha Göttert**: Data curation; methodology. **Noomen Hamed**: Methodology; resources. **Julius C. Fischer**: Resources. **Jürgen Ruland**: Resources. **Dirk H. Busch**: Resources. **Hendrik Poeck**: Resources. **Katharina S. Götze**: Resources. **Anna L. Illert**: Resources. **Florian Bassermann**: Project administration; supervision; resources; funding acquisition. **Simon M. Heidegger**: Supervision; conceptualization; investigation; funding acquisition; writing—original draft; writing—review and editing; resources.

## CONFLICT OF INTEREST STATEMENT

The authors declare no conflicts of interest.

## FUNDING

This study was supported by the German José Carreras Foundation (DJCLS 07R/2020 to S.H.), the Wilhelm Sander Foundation (2021.041.1 to S.H.; 2023.101.1 to N.E.K.), the European Hematology Association (RG‐202210‐02846 to N.E.K.), the Deutsche Forschungsgemeinschaft (SFB‐TRR 338/201‐45881907, project A01 to D.H.B.), the Deutsche Forschungsgemeinschaft (TRR 387/1 – 514894665 to F.B. and A.L.I.), the Mildred‐Scheel‐Professorship from the German Cancer Aid (DKH‐70114112) and EU funding (MSCA‐DN 101071735) to A.L.I., and the Technical University of Munich TranslaTUM Seed Fund to N.E.K. Open Access funding enabled and organized by Projekt DEAL.

## Supporting information

Supporting Information.

Supporting Information.

## Data Availability

The data that support the findings of this study are available from the corresponding author upon reasonable request.
