# Supplementary material for: Modulation of the RNA‐sensing RIG/MAVS pathway bypasses resistance to hypomethylating agents in acute myeloid leukemia
Source: Hemasphere. 2026 Nov 11;10(11):e70523. doi: 10.1002/hem3.70523 (PMC13753967; doi:10.1002/hem3.70523)

Supplementary Figure 1

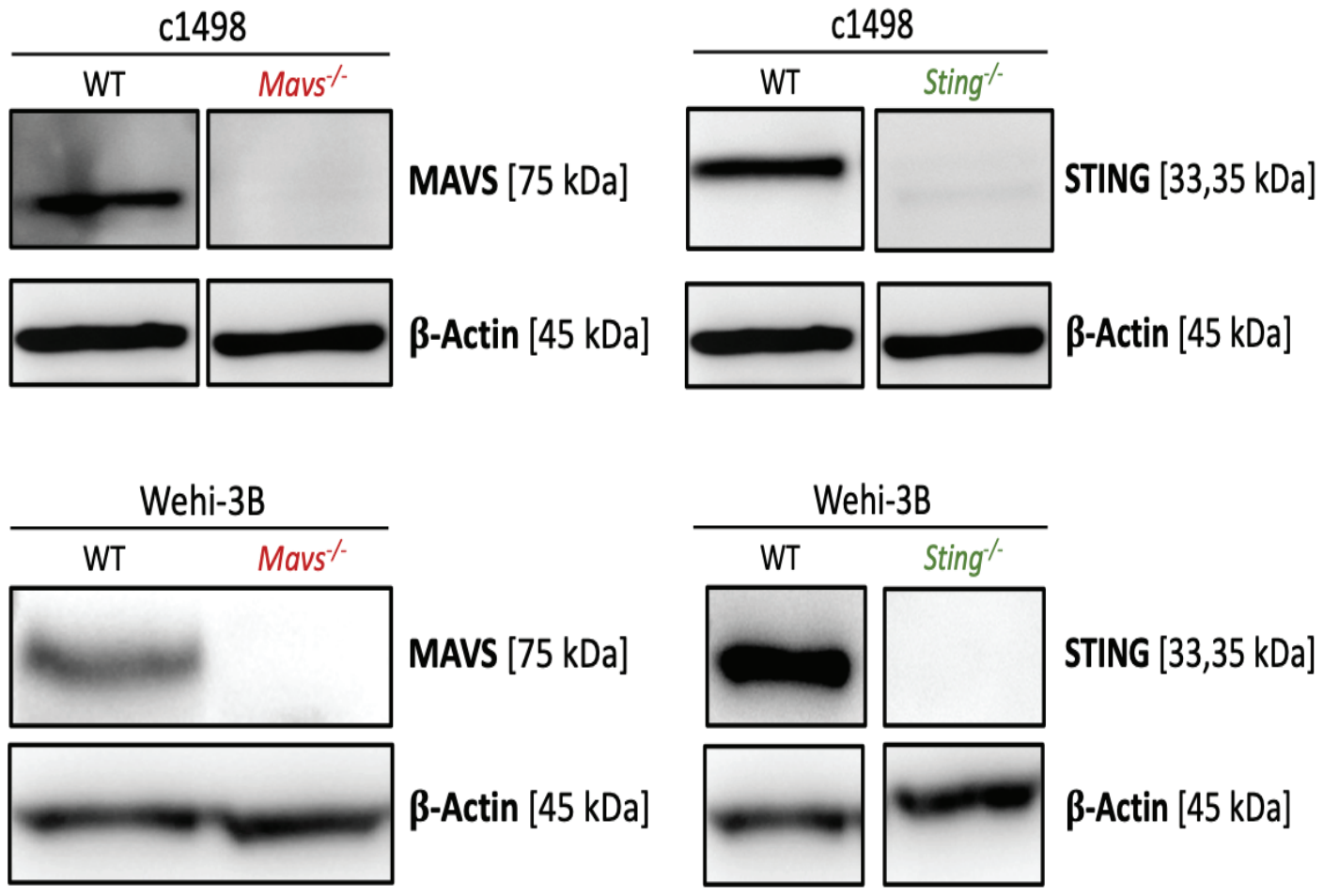

Supplementary Figure 2

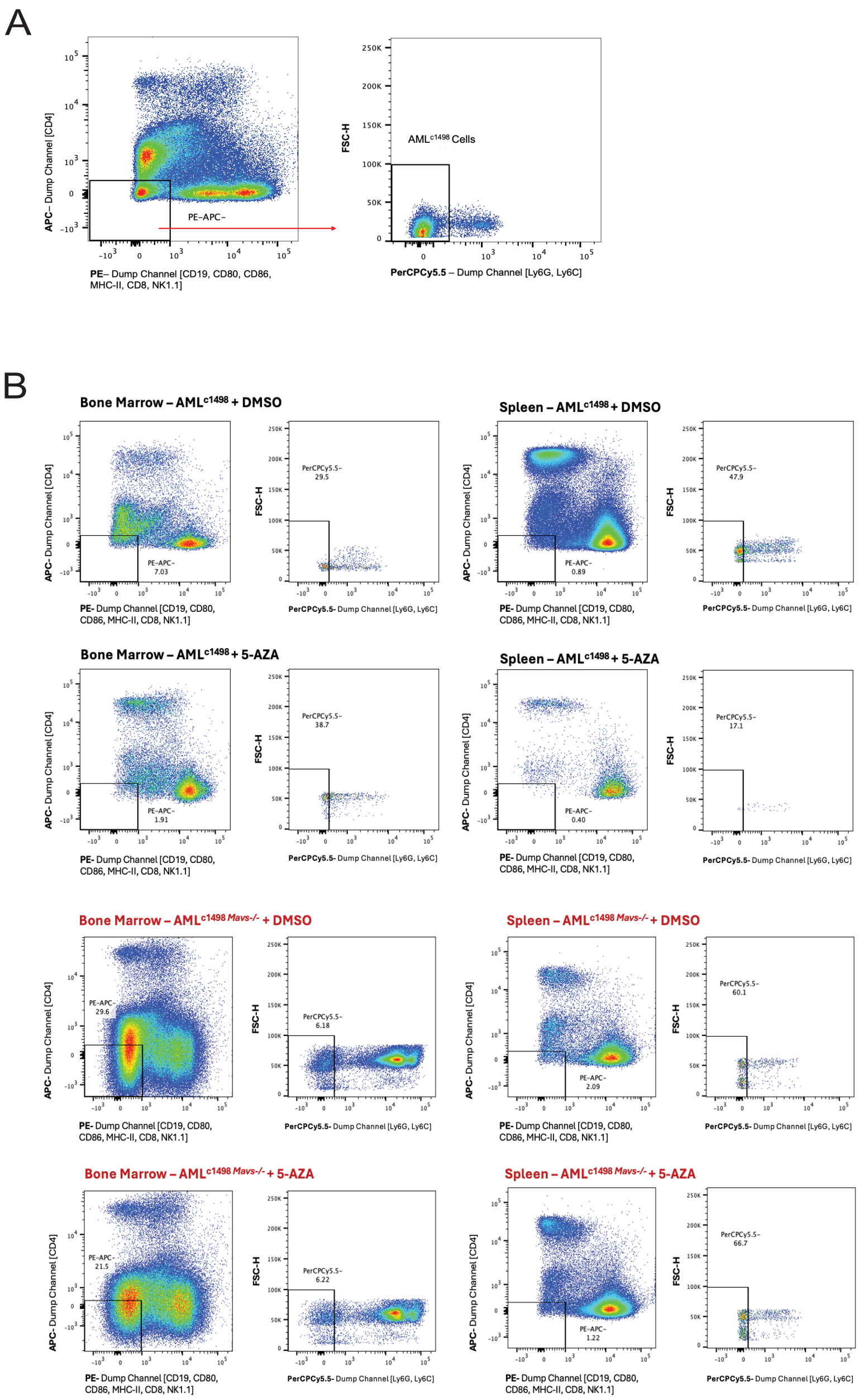

Supplementary Figure 3

A

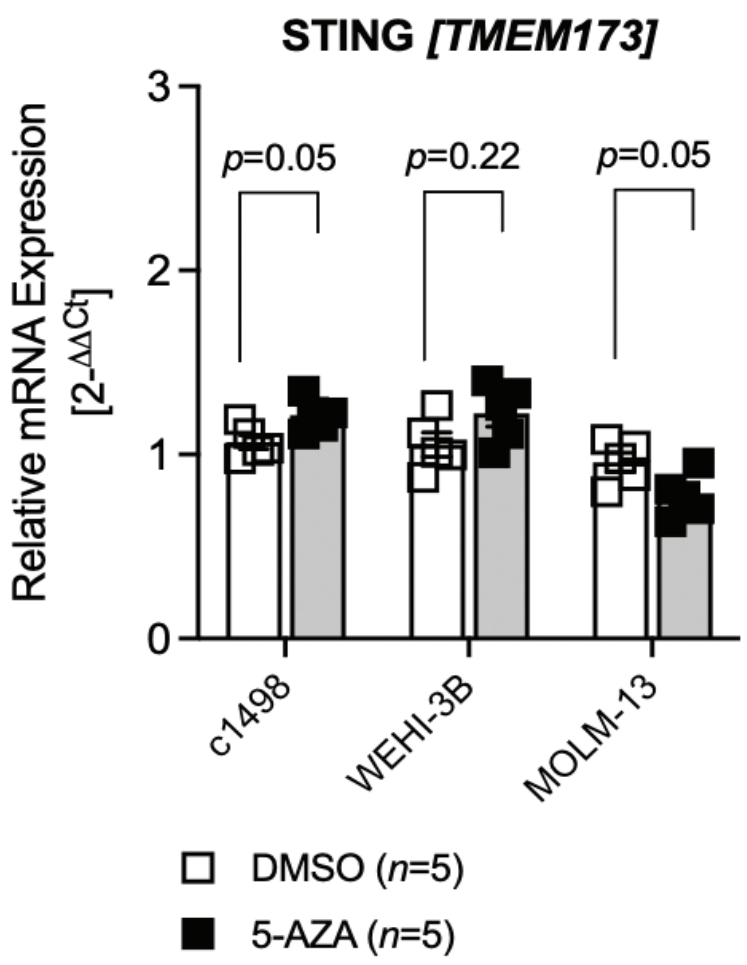

B

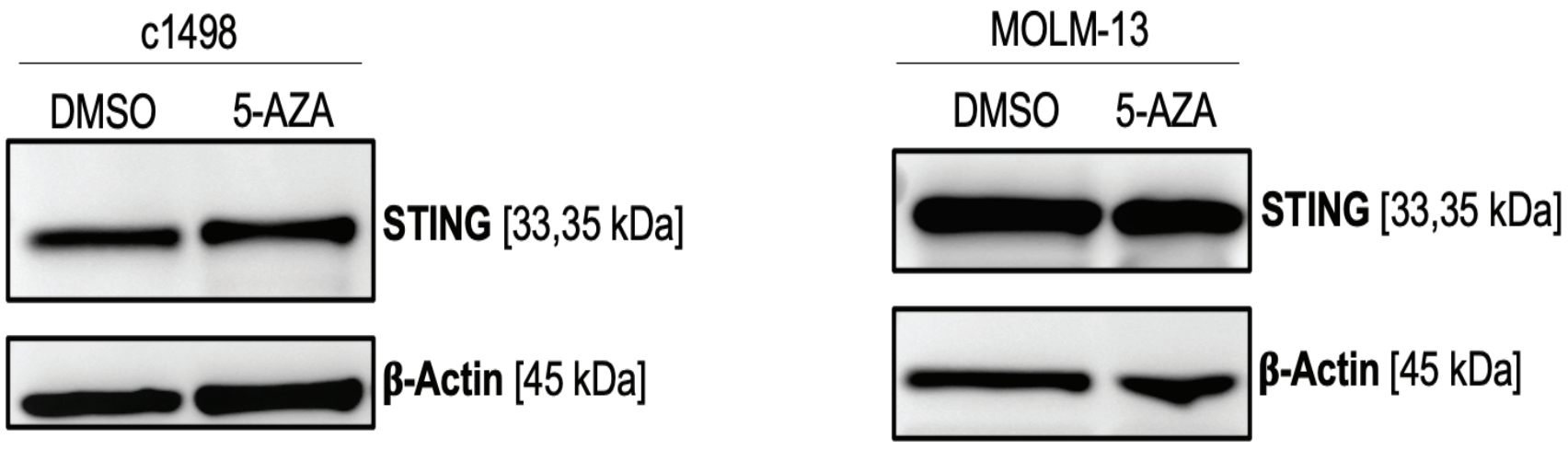

Supplementary Figure 4

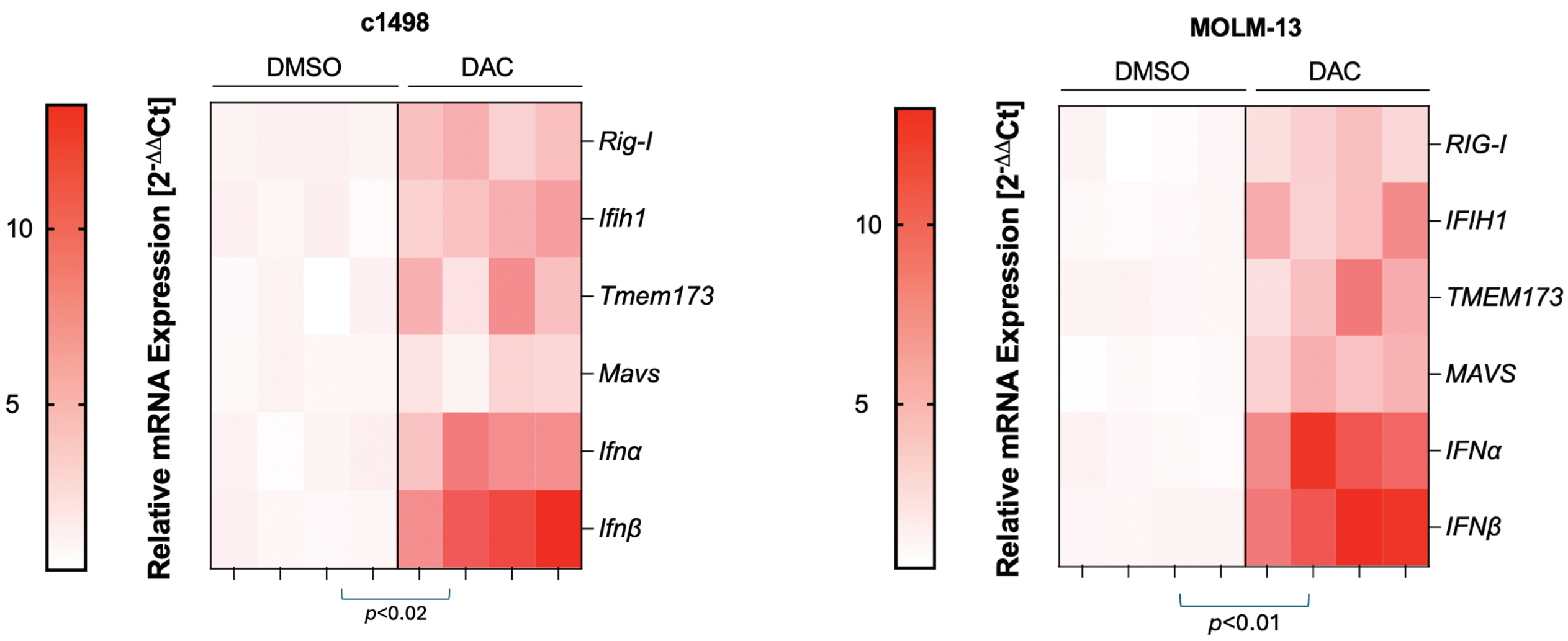

Supplementary Figure 5

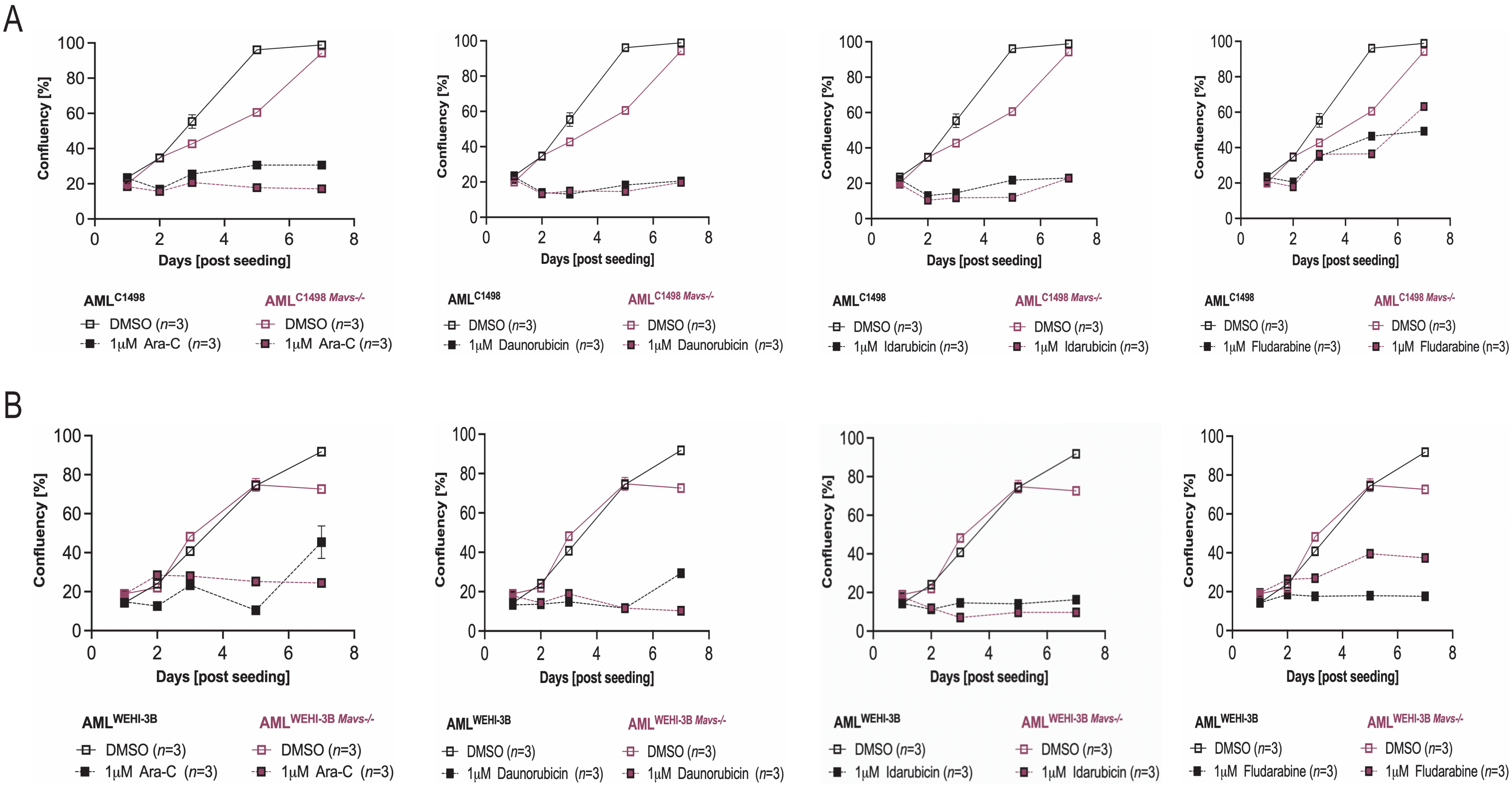

Supplementary Figure 6

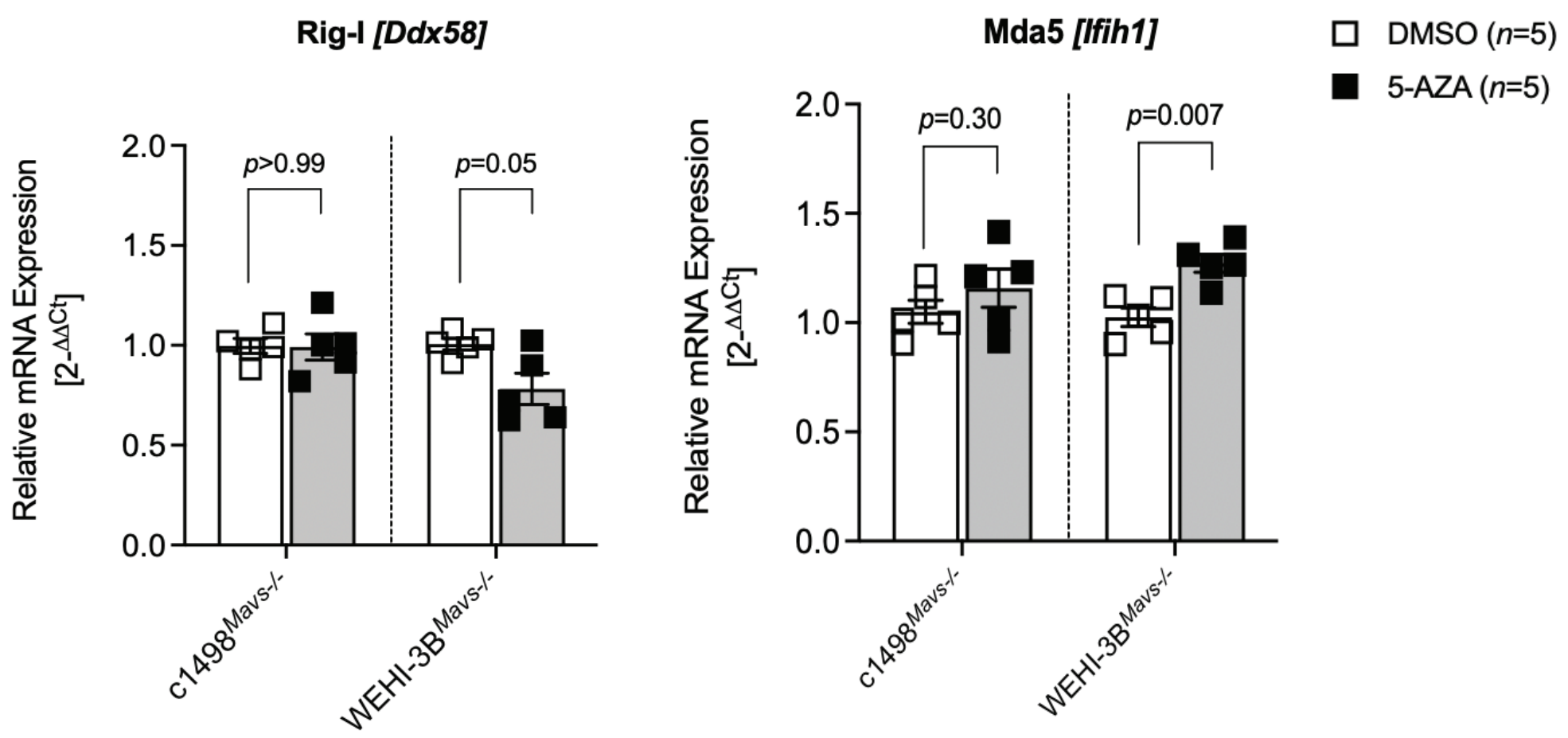

Supplementary Figure 7

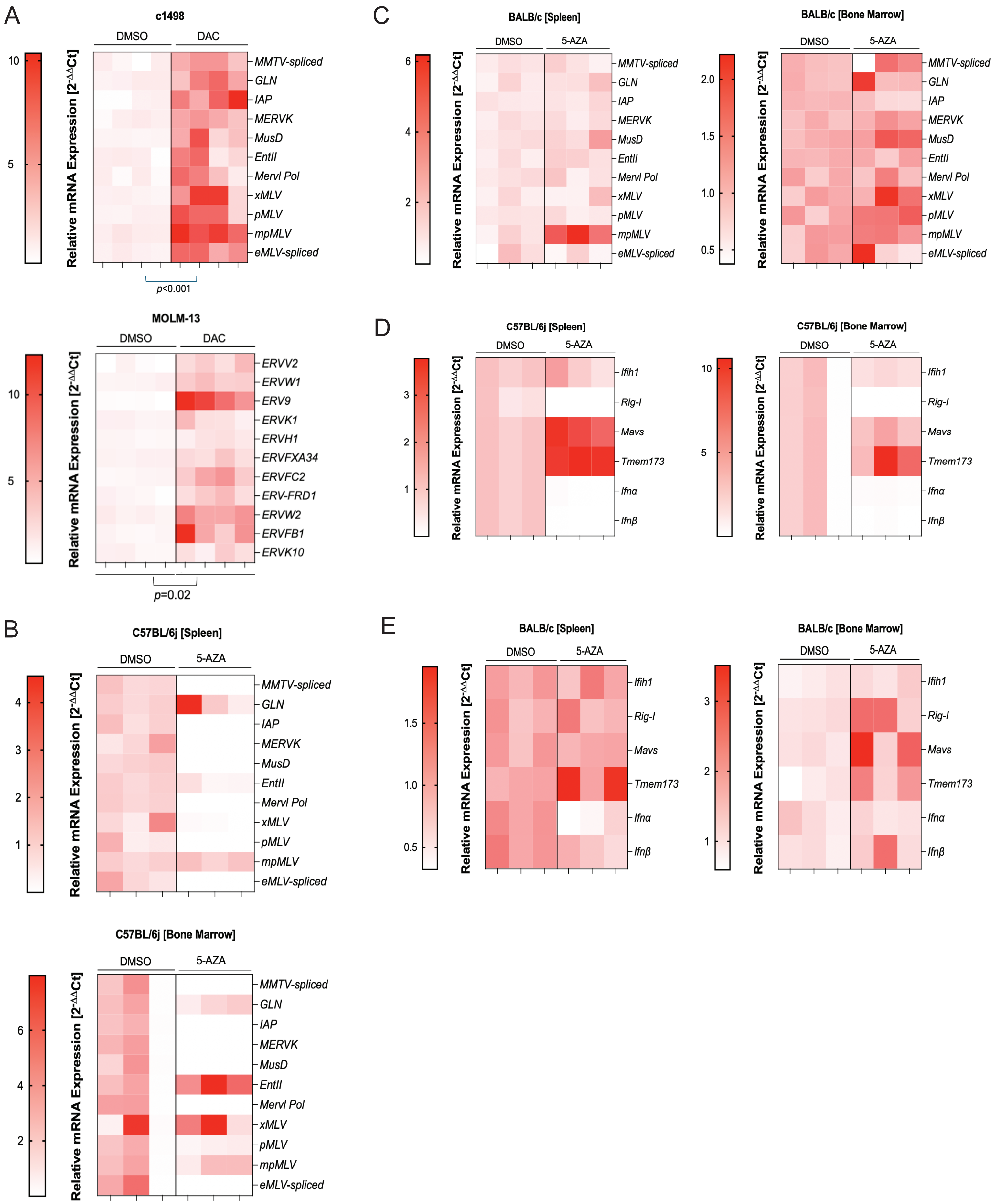

Supplementary Figure 8

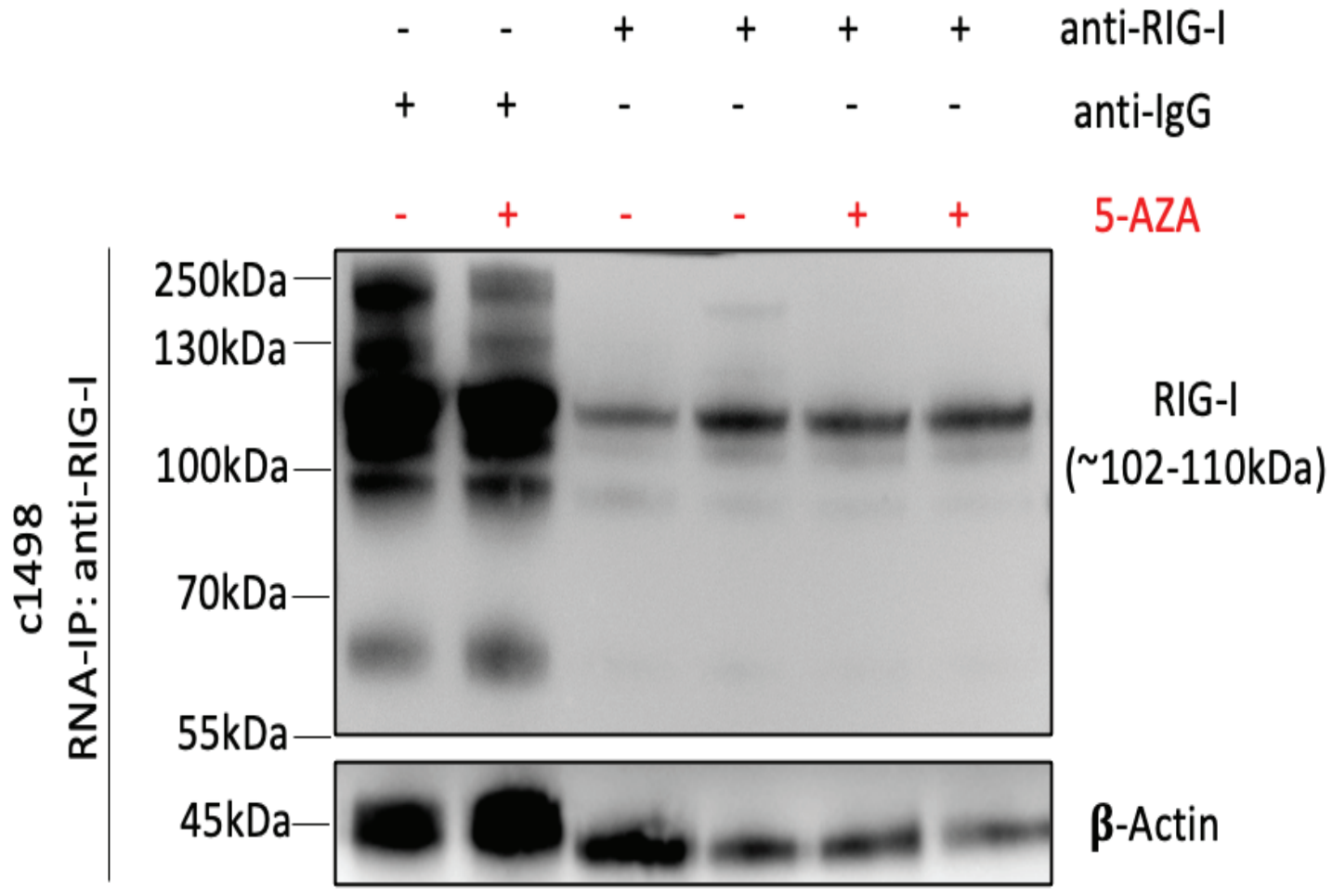

Supplementary Figure 9

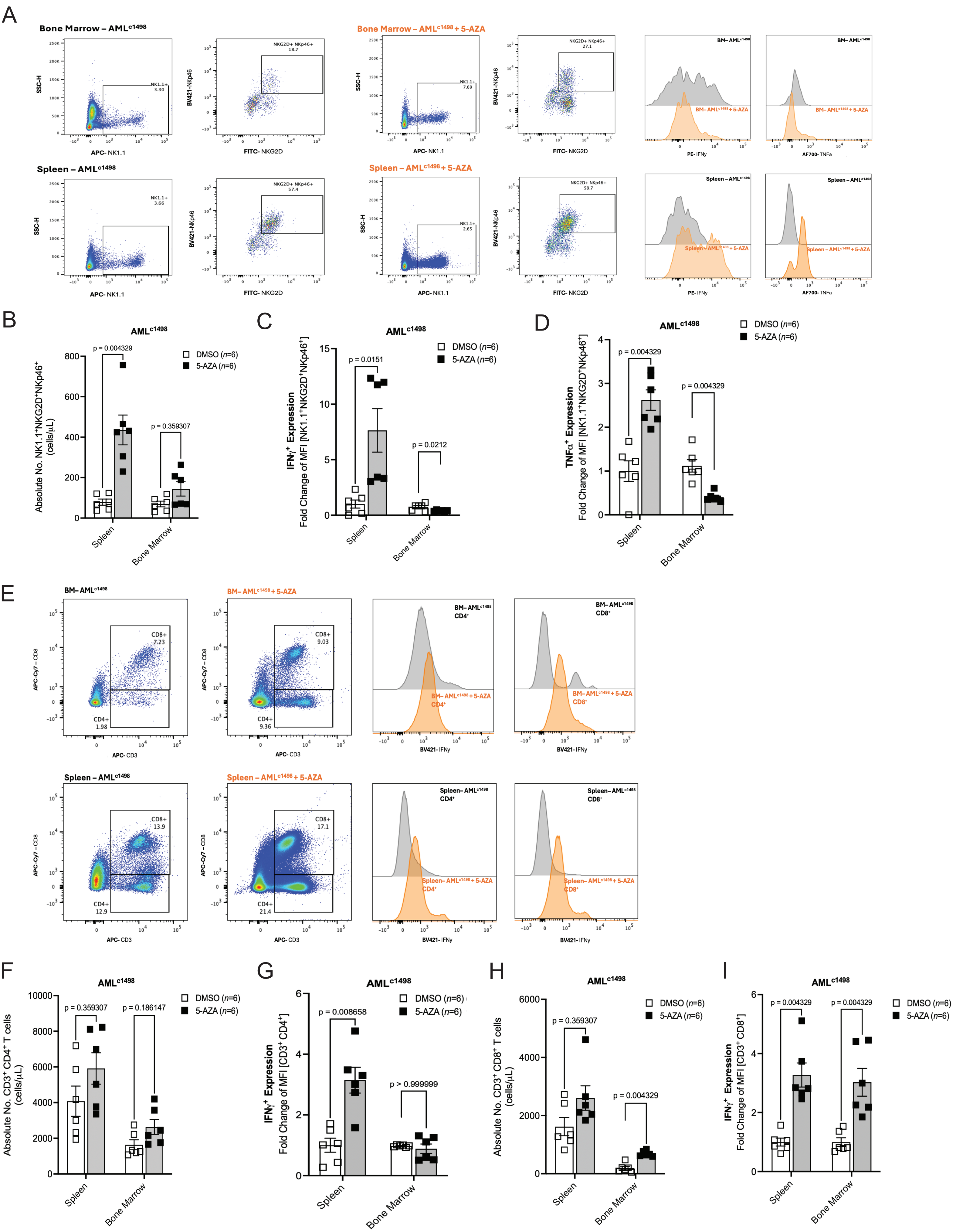

Supplementary Figure 10

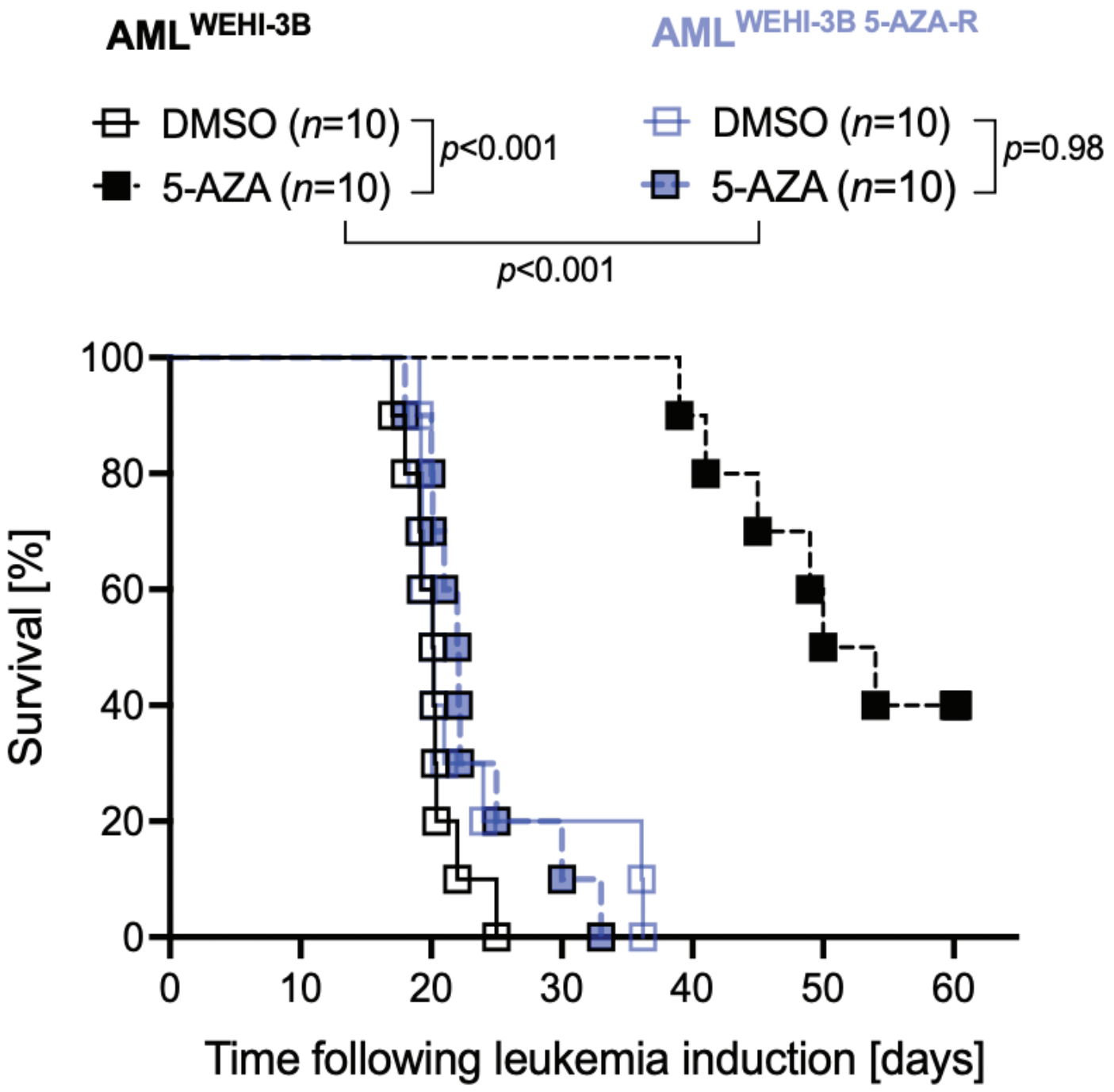

Supplementary Figure 11

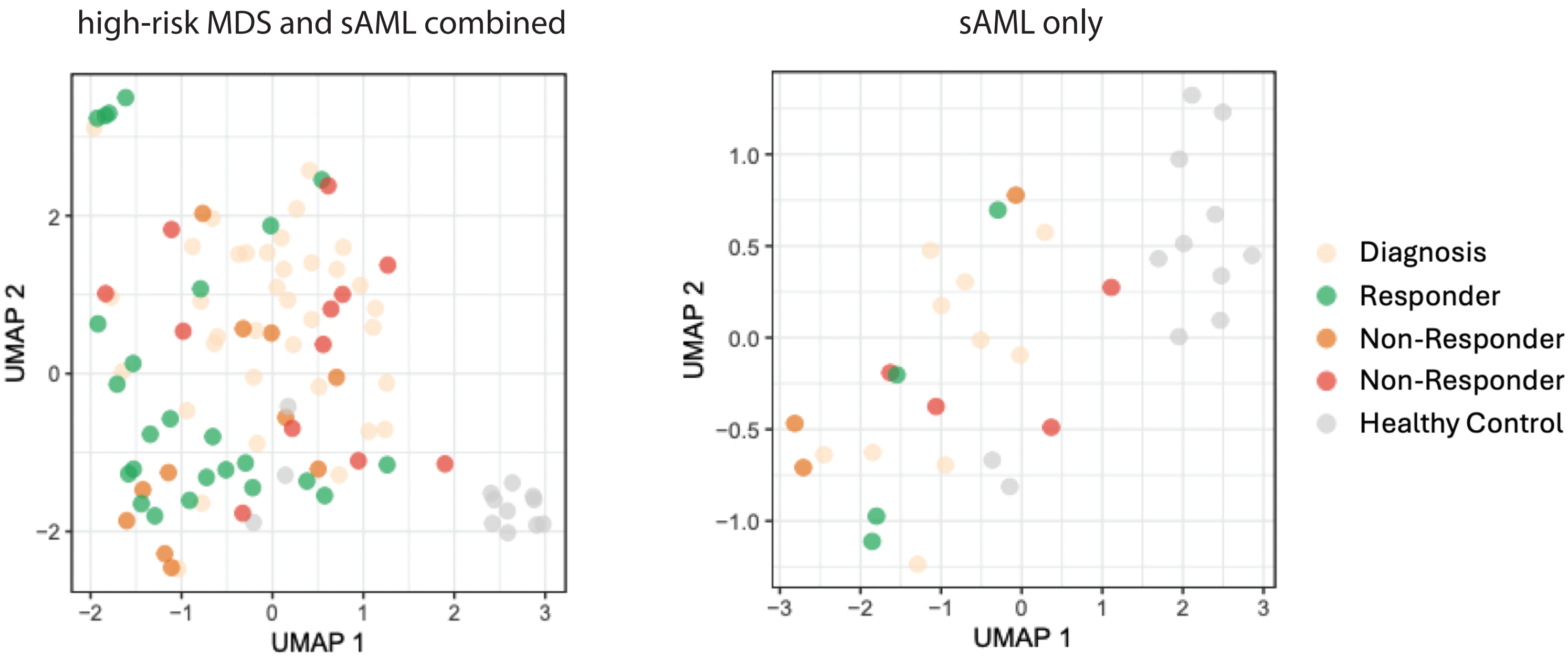

Supplement: Supplementary file 1 [file HEM3-10-e70523-s001.pdf]
