# Supplementary material for: Modulation of the RNA‐sensing RIG/MAVS pathway bypasses resistance to hypomethylating agents in acute myeloid leukemia
Source: Hemasphere. 2026 Nov 11;10(11):e70523. doi: 10.1002/hem3.70523 (PMC13753967; doi:10.1002/hem3.70523)
Supplement: Supplementary file 2 [file HEM3-10-e70523-s002.docx]

**SUPPLEMENTARY MATERIALS AND METHODS**

**Mice.** 5-7-week-old female C57Bl/6j and BALB/c mice were purchased from Janvier. Mice genetically deficient for the interferon-α and -β receptor subunit 1 (B6(Cg)-Ifnar1tm1.2Ees/J; RRID: IMSR_JAX:028,288; herein referred to as *Ifnar1^−/−^*) have been previously described (1). Wild-type and genetically deficient mice included in the same experiments were either littermates from heterozygous breeding or were co-housed for at least 4 weeks before onset of experiments. Mice were between 6 and 10 weeks old at the beginning of the experiments and were kept under specific pathogen-free (SPF) conditions in a controlled environment at the animal facilities of the University Hospital Regensburg or the TUM University Hospital, Munich. Mice were maintained on a 12h light–dark cycle, with 12h of light followed by 12h of darkness, a constant temperature of 24°C, and a humidity level of 55%–65%. Animal protocols were approved by the Bavarian animal ethics committee (Regierung von Oberbayern, Munich, Germany, ROB-55.2-2532.Vet_02-19-159).

**Leukemia cell lines and culture conditions.** Human and murine AML cell lines with distinct, well-characterized genomic profiles were utilized to model disease diversity and therapeutic response. (WEHI-3B) and human (MOLM-13) leukemia cell lines were purchased from ATCC (American Type Culture Collection, Manassas, Virginia, USA). MOLM-13 (derived from a patient with acute myelomonocytic leukemia, FAB subtype M5a) are genetically characterized by a hyper diploid karyotype carrying the aggressive FLT3-ITD mutation and the MLL-AF9 (t9;11) chromosomal translocation. The murine myelomonocytic leukemia cell line WEHI-3B (FAB subtype M4, BALB/c background) serves as a classic model for autocrine-driven oncogenesis resulting from genomic retroviral transpositions. Specifically, a retroviral Intracisternal A-Particle (IAP) insertion proximal to the Il3 gene promoter forces constitutive Interleukin-3 production, which acts cooperatively with a proviral integration-induced over-activation of the Hoxb8 homeobox gene to block terminal differentiation and fuel leukemia progression. The murine C1498 AML cell line was kindly provided by Lars Koenig (Ludwig-Maximillian University, Munich, Germany). Genomic profiling reveals that C1498 cells exhibit a complex molecular landscape driven by deleterious mutations in Trp53 and Tet2, alongside a major Myc amplification and structural deletions within the cohesin complex gene network (2). The WEHI-3B and MOLM-13 cell lines were cultured in RPMI-1640 supplemented with 10% (v/v) FCS (Gibco), 3 mM L-glutamine, 100 U/ml penicillin, 100 μg/ml streptomycin (Sigma Aldrich), while C1498 cells were cultured in DMEM media containing 10% (v/v) FCS, 3 mM L-glutamine, 100 U/ml penicillin, 100 μg/ml streptomycin, and 50 μM β-mercaptoethanol (Gibco). All cell lines were routinely tested for mycoplasma and validated using STR analysis with 18 STR markers.

**Generation of 5-Azacytidine-resistant leukemia cell lines.** The IC50 of all three cell lines was first determined by titrating the cells in increasing concentrations of 5-AZA. Each cell line was cultured for 48-72h in the determined concentration that would yield approximately 50% cell death. Individually growing cells were obtained, propagated in increasing concentrations of 5-AZA over at least 30 days. Resistance to 5-AZA was confirmed functionally (lack of cell death at highly cytotoxic concentrations) and via Western Blot (inability to suppress expression of DNMTs compared to the 5-AZA sensitive (WT) version of the cell lines). Resistant stocks of each cell line were frozen at -80^o^C until use. Prior to experiments, resistant AML cells were thawed and cultured once in a maintenance dose of 5-AZA (1μM) for one week and taken off maintenance treatment 4-5 days before the commencement of downstream experiments.

**All-*trans*-retinoic acid (ATRA) treatment of leukemia cell lines *in vitro* and *in vivo.*** ATRA (Sigma Aldrich) was reconstituted to a stock solution of 40mg/mL in DMSO. Initial titration experiments were carried out in WT and AZA resistant (5-AZA-R) AML cell lines with concentrations ranging from 100nM - 1µM. Strong cell death activity of ATRA was observed at 100nM and was used for all subsequent experiments. For *in vivo* experiments, WT or 5-AZA-R WEHI-3B were inoculated into BALB/c mice as described above. On day 3, mice were randomized into groups to receive i.p. either PBS (100μL), or ATRA (2.5mg/kg in 100μL; eqv. 100nM *in vitro*). Injections were conducted daily from day 3-19 to best reflect the clinical dosing schedule in patients. Endpoint and survival analysis of mice were conducted as described above. For re-challenge experiments, ATRA treated 5-AZA-R AML bearing mice that survived until day 60 were re-inoculated with 1x10^6^ WEHI-3B 5-AZA-R murine AML cells.

**Conventional Flow cytometry.** All antibodies used for flow cytometry were purchased from BioLegend, eBioscience or BD Biosciences and are listed in the Supplementary Table S3. Briefly, cells were prepared as a single-cell suspension, washed twice with ice-cold PBS, and stained with a mix of Live/Dead Aqua viability dye (1:500) and CD16/CD32 Fc Block (1:50) for 20 min at 4°C. Extracellular markers (1:200) were stained for 45 min on ice in the dark. After washing, cells were resuspended in 200µL FACS buffer (PBS + 2mM EDTA, 2% FCS, 5% sodium azide). For intracellular staining, cells were incubated for 3-4h in RPMI with a cell stimulation cocktail and protein transport inhibitor (Thermo Fisher), then processed using the Foxp3 Transcription Factor Fixation/Permeabilization kit (eBioscience). For apoptosis analysis, cells were stained with Annexin V (BioLegend) in Annexin V binding buffer for 15 min in the dark, followed by propidium iodide (BioLegend) without washing. Data were acquired using FACS Canto II or LSRFortessa (BD Biosciences) and analyzed with FlowJo v10.9/v10.10 (Treestar, Ashland, OR).

**Mass Cytometry**

**Heavy-metal conjugation of antibodies.** Prior to heavy-metal conjugation, buffer exchange was performed for carrier-free formulated antibodies using Amicon 10K buffer exchange columns (Millipore). Following a 20min centrifugation at 14,000 x *g*, 4°C, another washing step was performed with PBS for 15min at 14,000 x *g*, 4°C before the buffer-exchanged antibody was collected for subsequent custom labelling. Label-free antibodies – targeting selected key metabolic enzymes – were conjugated to heavy-metal ions with commercially available Maxpar X8 resp. MCP9 labeling reagents (Standard BioTools) according to the manufacturer’s protocol. Resulting protein concentration was determined spectrophotometrically using NanoDrop (Thermo Fisher) and antibody stabilization buffer (Candor Bioscience) was added to a final concentration of 0.5 mg mL^-1^. Custom-labeled antibodies were validated for optimal staining using lymph node (LN) T cells after being stimulated overnight with 5µg mL^-1^ plate-bound purified anti-CD3 clone 145-2C11 and 2µg mL^-1^ soluble purified anti-CD28 clone 37.51 antibodies (both BioLegend).

**Cell staining.** Mass cytometric staining was performed at RT. Cells were resuspended in serum-free complete RPMI-1640 (Thermo Fisher) and labeled with a final concentration of 5µM Cisplatin-195Pt (Standard BioTools) for 5min to discriminate live/dead cells. Cells were washed in Maxpar Cell Staining Buffer (CSB) (Standard BioTools) and blocked with anti-CD16/32 clone 93 (1:100) and anti-CD16.2 clone 9E9 (1:100) (both BioLegend) in CSB for 10min, before adding cell surface antibody mix in CSB for 30min. Cells were then fixed and permeabilized using the Foxp3 staining kit (eBioscience). Limiting technical variability during staining and data acquisition, individual samples within one experiment were palladium-barcoded using the CellID^TM^ 20-Plex Pd Barcoding Kit (Standard BioTools) according to the manufacturer’s instruction and combined into a composite sample before further processing. Intracellular staining was performed by adding the intracellular antibody mix in 1x perm/wash buffer (eBioscience) for 1h. After two consecutive wash steps in 1x perm/wash buffer and CSB, cells were resuspended in intercalation solution (4% PFA and 125nM CellID^TM^ Intercalator-Ir (Standard BioTools) in Maxpar Fix and Perm Buffer (Standard BioTools) rotating overnight at 4°C. Before acquisition, samples were washed once in CSB and twice
in Cell Acquisition Solution (CAS) (Standard BioTools) and then resuspended at
1x10^6^ cells mL^-1^ in CAS supplemented with 1x EQ^TM^ Four Element Calibration Beads (Standard BioTools) and acquired on a Helios^TM^ Mass Cytometer (Standard BioTools) with the following parameters: lower convolution threshold 400, event length 10–150, sigma 3, signal subtraction 0, event rate of 200–350 events/sec. Markers used for mass cytometric cell staining are provided in Supplementary Table S4.

**Mass cytometry data pre-processing and analysis.** Raw mass cytometry data was normalized and debarcoded using the Standard BioTools software (Minimum barcode separation = 0.2, Maximum Mahalanobis Distance = 10.0). Normalized FCS files were imported into Flowjo (BD Biosciences) and gated according to a gaussian parameter clean-up (Approach to Bivariate Analysis of Data Acquired Using the Maxpar Direct Immune Profiling Assay), which assures exclusion of noise, technical artifacts and ion fusion events. CD45^+^ immune cells were pre-gated further and imported – as an exported fcs-file – into R Studio. Single-cell mass cytometry data from spleen and bone marrow samples were preprocessed using the CATALYST workflow and arcsinh-transformed with a cofactor of 5, followed by downsampling to the minimum number of cells per sample. Lineage and functional marker panels were defined, and dimensionality reduction was performed using UMAP on lineage markers to visualize cellular heterogeneity. Cells were clustered using FlowSOM followed by ConsensusClusterPlus metaclustering, and clusters were annotated to canonical cell types based on marker expression profiles. Differential abundance (DA) and differential state (DS) analyses were conducted using diffcyt with edgeR and limma, respectively, to identify significant changes in cluster proportions between conditions. All analyses were performed in R 4.4.1 on Ubuntu 22.04.3 LTS.

**Cell proliferation under cytotoxic stress.** Cells were seeded at a density of 5000 cells/well in 96-well plates. WT or MAVS-deficient WEHI-3B or C1498 AML cells were treated with 1μM (following initial titration experiments) DNA-damaging agents (cytarabine, daunorubicin, fludarabine and idarubicin). Percentage of cell confluence was determined every 24h by automated bright-field microscopy using a Celigo Image Cytometer (Nexcelom). Media was exchanged every 48h supplemented with the relevant drugs or DMSO. Cell proliferation was calculated using the Celigo software from the increase in plate confluency of technical triplicates at each time point.

**RNA isolation and quantitative real-time PCR.** RNA from cell lines and primary AML samples was isolated using the innuPREP RNA Mini kit 2.0 according to the manufacturer’s instructions (iST Innuscreen). RNA concentration and quality were determined by Nanodrop measurement and RNA was stored at -80^o^C until use. For reverse transcription, at least 200-1000ng of RNA was used and reverse transcription was performed with the High-Capacity cDNA Kit (Applied Biosystems, Thermo Fisher Scientific) according to the manufacturer’s instructions. Real-time quantitative PCR (qPCR) was performed using the PowerUp SYBR Green Master Mix (Thermo Fisher) on the QuantStudio (Thermo Fisher) with 40 cycles of amplification according to the manufacturer’s instructions. The ΔΔCt method was employed to analyse the raw data, with normalization to the murine or human β-Actin housekeeping gene. The murine and human primer sequences used in the manuscript are listed in Supplementary Table S5.

**Western blotting.** Cell lysates were generated using RIPA lysis buffer (Thermo Fisher) supplemented with phosphatase and protease inhibitor mixture (Sigma Aldrich). The total protein concentration of each sample was determined using the Pierce BCA Protein Assay Kit (Life Technologies). The samples were run, and total proteins were resolved using 4-10% sodium dodecyl sulphate-polyacrylamide electrophoresis gels (NuPAGE, Invitrogen). The proteins from the gel were transferred onto PVDF membranes (Perkin Elmer) and blocked for 1h with 5% bovine serum albumin in 1x Tris buffer saline containing 0.1% tween-20 (TBS-T) (Thermo Fisher). The membranes were subsequently incubated with primary antibodies in their respective blocking solution overnight at 4^o^C on a rocking platform. Depending on the primary antibody, secondary antibody incubation was performed with anti-rabbit or anti-mouse IgG HRP-linked antibodies. The membranes were visualized using the Pierce SuperSignal West Pico PLUS or Femto Chemiluminescent Substrate (Thermo Fisher) and captured using the Bio-Rad ChemiDoc MP. All proteins were detected on the same membrane for each western blot figure throughout the manuscript. All images were processed and quantified using ImageJ software. Individual phosphoprotein signals were normalized directly to their respective total target protein levels to calculate relative activation status. Total target protein levels were normalized directly to their respective β-Actin loading control. To determine the change in protein expression between conditions, fold change of 5-AZA was calculated relative to the DMSO controls for each cell line. In some cases, western blot images were cropped to highlight representative replicates from the main membrane. Black borders or dotted lines indicate non-adjacent lanes were separated to improve clarity while preserving data integrity. The antibodies used for Western blotting are listed in Supplementary Table S6.

**DNA Methylation Data Analysis.** Publicly available Illumina Infinium HumanMethylation450K array data from AML/MDS patients and healthy controls were downloaded from GEO (accession GSE152710) and reanalyzed (Supplementary Table 7). Raw IDAT files were imported using the *minfi* package (R/Bioconductor). Sample-level quality control included assessment of detection p-values, median signal intensities, and predicted sex. Background correction was performed using the NOOB (normal-exponential out-of-band) method. Probes were filtered for poor detection (p > 0.01 in ≥10% of samples), known SNP overlap, cross-reactive sequences, and sex chromosome location, yielding a final set of high-confidence autosomal probes. Pre-processed beta values were used for all downstream analyses. For global methylation analysis, dimensionality reduction was performed by UMAP on the 10,000 most variable probes. Targeted pathway analysis focused on gene body probes (Body and 3'UTR annotations) of curated gene sets comprising the RIG-I/MAVS signaling pathway, IFN-stimulated genes, and ERV regulators, only genes with a gene body coverage of ≥3 probes were retained. Sample-level pathway methylation was averaged per gene and unsupervised clustering (hierarchical cluster of beta values with n = 5 clusters) was applied to stratify patients. Cluster assignments were related to clinical variables (disease type, treatment, response) by frequency heatmaps and Fisher's exact tests. ERV methylation was quantified by intersecting 450K probe coordinates with RepeatMasker annotations (hg19; AnnotationHub) for three ERV families (ERV1, ERVK and ERVL). Only methylated ERV elements (average beta > 0.2) were considered and included in our analysis. Family-level methylation was computed as the mean beta across overlapping probes per sample and compared against a permutation-based random background (100 resampling iterations matched for probe count) to control for global methylation shifts. Methylation levels for samples with multiple follow-ups were normalized to the value at diagnosis. Differences across clinical groups were tested using Kruskal-Wallis tests with Benjamini-Hochberg correction. All analyses were performed in R using tidyverse, limma, minfi, umap, pheatmap, and GenomicRanges packages, with a fixed random seed (42) for reproducibility. All Code used for analysis and figure generation was deposited on github. The repository will be made public upon publication.

**Supplementary Table 1: AML Patient demographics and treatment characteristics**

| **Patient ID** | **Gender/Age** | **Treatment Response** | **Treatment** | **Blast Percentage** | **Time between diagnosis and follow up sample acquisition** |
| --- | --- | --- | --- | --- | --- |
| 1 | M/80 | Y | 12 cycles of 5-AZA | 64% – At Diagnosis  <5% – Remission | 12 months |
| 2 | F/73 | Y | 4 cycles of 5-AZA | 32% – At Diagnosis  <5% – Remission | 6 months |
| 3 | M/85 | Y | 6 cycles of 5-AZA | 23% - At Diagnosis  <5% - Remission | 6 months |
| 4 | M/78 | Y | 9 cycles of Ven/5-AZA | 33% - At Diagnosis  <5% - Remission | 12 months |
| 5 | M/84 | N | 8 cycles of Ven/5-AZA | Unknown | 14 months |
| 6 | F/76 | N | 2 cycles of Ven/5-AZA | 80% - At Diagnosis  40% - Refractory | 2 months |
| 7 | M/87 | N | 5 cycles of Ven/5-AZA | 70% - At Diagnosis  25% - Relapse | 5 months |

**Supplementary Table 2: AML CRISPR-Cas-mediated genome editing guide RNA target sequences and Sanger sequencing primers**

| **Gene** | **Target sequences** |
| --- | --- |
| *Mavs* (murine) | gRNA1: 5’- CCGGTTCCCGATCTGCCTGT -3’  gRNA2: 5’- TCTTCAATAATCTCCAGCGC -3’ |
| *Rig-I* (murine) | gRNA1: 5’- TGTAGCTGAGGATGTAGGTG -3’  gRNA2: 5’- CCTGGCTCGAGGATGGTGAG -3’ |
| *Sting* (murine) | gRNA1: 5’-TTGCCACCCAAAGGATCATC-3’  gRNA2: 5’-CACCTAGCCTCGCACGAACT-3’ |
|  | **Sanger sequencing primers** |
| *Mavs* (murine) | Fwd: 5’-TGCCTGCAAACCTTGATGTG-3’  Rev: 5’-AGGGAAAGTGACTGGGTGTAG-3’ |
| *Rig-I* (murine) | Fwd: 5’-CCCTGAGGCAGTTTCGATTT-3’  Rev: 5’-ACAGGAATCTAAGGAGGGGC-3’ |
| *Sting* (murine) | Fwd: 5’-TAGTCAGGACCTTAGGCCCT-3’  Rev: 5’-CCATAGTCCCCTCTTCCCAC-3’ |

**Supplementary Table 3: Antibodies for flow cytometry**

| **Antibody** | **Clone** | **Catalogue number** | **Fluorochrome** | **Vendor** |
| --- | --- | --- | --- | --- |
| NK1.1 | PK136 | 108710 | APC | BioLegend |
| NK1.1 | PK136 | 108707 | PE | BioLegend |
| NKG2D (CD314) | C7 | 115711 | FITC | BioLegend |
| NKp46 (CD335) | 29A1.4 | 137611 | BV421 | BioLegend |
| CD69 | H1.2F3 | 104520 | PerCP | BioLegend |
| IFNγ | XMG1.2 | 505808 | PE | BioLegend |
| TNFα | MP6-XT22 | 506338 | AF700 | BioLegend |
| Granzyme B | NGZB | 25-8898-82 | PeCy7 | eBioscience |
| CD45 | 30-F11 | 103128 | AF700 | BioLegend |
| CD45 | 30-F11 | 103125 | Pacific Blue | BioLegend |
| CD45.2 | 104 | 109829 | PeCy7 | BioLegend |
| CD3 | 17A2 | 100204 | FITC | BioLegend |
| CD3 | 17A2 | 100236 | APC | BioLegend |
| CD4 | GK1.5 | 17-0041-82 | APC | eBioScience |
| CD8 | 53-6.7 | 100734 | PerCPCy5.5 | BioLegend |
| CD8 | 53-6.7 | 100714 | APC-Cy7 | BioLegend |
| CD8 | 53-6.7 | 12-0081-81 | PE | eBioscience |
| CD44 | IM7 | 103028 | APC-Cy7 | BioLegend |
| CD62L | MEL-14 | 12-0621-83 | PE | eBioscience |
| CD25 | PC61 | 102006 | FITC | BioLegend |
| CD11b | M1/70 | 101226 | APC-Cy7 | BioLegend |
| CD11b | M1/70 | 101212 | APC | BioLegend |
| CD11c | 17-0114-82 | 17-0114-82 | APC | eBioscience |
| CD11c | N418 | 117306 | FITC | BioLegend |
| MHC-I | AF6-88.5.5.3 | 48-5958-82 | Pacific Blue | eBioscience |
| MHC-II | M5/114.15.2 | 107606 | FITC | BioLegend |
| MHC-II | M5/114.15.2 | 107607 | PE | BioLegend |
| F4/80 | BM8 | 25-4801-82 | PeCy7 | eBioscience |
| CD80 | 16-10A1 | 104724 | Pacific Blue | BioLegend |
| CD80 | 16-10A1 | 104708 | PE | BioLegend |
| CD86 | GL-1 | 105016 | PeCy5 | BioLegend |
| CD86 | GL-1 | 105014 | PeCy7 | BioLegend |
| CD86 | GL-1 | 12-0862-82 | PE | eBioscience |
| iNOS | W16030C | 696805 | PE | BioLegend |
| Ly6C | HK1.4 | 128011 | PerCP | BioLegend |
| Ly6G (Gr-1) | RB6-8C5 | 108428 | PerCPCy5.5 | BioLegend |
| IL-1β | NJTEN3 | 17-7114-80 | APC | eBioscience |
| CD103 | 2E7 | 121415 | PerCPCy5.5 | BioLegend |
| CD40 | 3/23 | 124611 | APC | BioLegend |
| CD19 | 1D3/CD19 | 152408 | PE | BioLegend |

**Supplementary Table 4: Panel for Mass Cytometry**

| **Label** | **Target** | **Clone** | **Manufacturer** | **Catalog number** |
| --- | --- | --- | --- | --- |
| 089Y | CD45 | 30-F11 | Standard BioTools | 3089005B |
| 112Cd | CD44 | IM7 | BioLegend | 103002 |
| 114Cd | PD-1 | J43 | ThermoFisher | 14-9985-82 |
| 141Pr | Ly-6G | 1A8 | Standard BioTools | 3141008B |
| 142Nd | ACLY | 4D11 | Invitrogen | MA524861 |
| 143Nd | CD69 | H1.2F3 | Standard BioTools | 3143004B |
| 144Nd | GAPDH | 6C5 | Invitrogen | AM4300 |
| 145Nd | CD4 | RM4-5 | Standard BioTools | 3145002B |
| 146Nd | CD8a | 53-6.7 | Standard BioTools | 3146003B |
| 147Sm | CD103 | 2E7 | BioLegend | 121402 |
| 148Nd | CD11b (Mac-1) | M1/70 | Standard BioTools | 3148003B |
| 149Sm | CD19 | 6D5 | Standard BioTools | 3149002B |
| 150Nd | Ly-6C | HK1.4 | Standard BioTools | 3150010B |
| 151Eu | CD25 (IL-2R) | 3C7 | Standard BioTools | 3151007B |
| 152Sm | CD3e | 145-2C11 | Standard BioTools | 3152004B |
| 153Eu | GLUT1 | EPR3915 | abcam | ab196357 |
| 154Sm | CD152 (CTLA-4) | UC10-4B9 | Standard BioTools | 3154008B |
| 155Gd | ACC | C83B10 | CellSignaling | 3676 |
| 156Gd | MHC-I | AF6-88.5 | BioLegend | 116502 |
| 158Gd | FoxP3 | FJK-16s | Standard BioTools | 3158003A |
| 159Tb | ATP5A | 7H10BD4F9 | abcam | ab110273 |
| 160Gd | CD62L (L-selectin) | MEL-14 | Standard BioTools | 3160008B |
| 161Dy | Tbet | 4B10 | Standard BioTools | 3161014B |
| 162Dy | TNFa | MP6-XT22 | Standard BioTools | 3162002B |
| 163Dy | F4/80 | BM8 | BioLegend | 123143 |
| 164Dy | CPT1A | 8F6AE9 | abcam | ab128568 |
| 165Ho | IFNg | XMG1.2 | Standard BioTools | 3165003B |
| 166Er | HK I | EPR10134(B) | abcam | ab150423 |
| 168Er | Ki-67 | B56 | Standard BioTools | 3168007B |
| 169Tm | G6PDP | EPR20668 | abcam | ab210702 |
| 170Er | CD161 (NK1.1) | PK136 | Standard BioTools | 3170002B |
| 171Yb | LDHA | EP1566Y | abcam | ab219591 |
| 172Yb | Perforin | OMAK-D | Standard BioTools | 3172018B |
| 173Yb | CD40 | 3/23 | BioLegend | 124602 |
| 174Yb | MHCII | M5/114.15.2 | Standard BioTools | 3174003B |
| 175Lu | CD86 | GL-1 | BioLegend | 105002 |
| 176Yb | CD278 (ICOS) | 7E.17G9 | Standard BioTools | 3176014B |
| 209Bi | CD11c | N418 | Standard BioTools | 3209005B |

Used markers for mass cytometric cell staining were either from Standard BioTools or purchased from Abcam, CellSignaling, BioLegend or Invitrogen (as indicated), before being custom-labeled using Maxpar X8 resp. MCP labeling reagents (Standard BioTools).

**Supplementary Table 5: Quantitative real-time PCR primer sequences**

| **Gene** | **Species** | **Target sequences** |
| --- | --- | --- |
| *Actb (β-actin)* | murine | Forward: 5’-CATTGCTGACAGGATGCAGAAGG-3’ Reverse: 5’-TGCTGGAAGGTGGACAGTGAGG-3’ |
| *Rig-I (Ddx58)* | murine | Forward: 5’-AGCCAAGGATGTCTCCGAGGAA-3’  Reverse: 5’-ACACTGAGCACGCTTTGTGGAC-3’ |
| *Mavs* | murine | Forward: 5’-CTGCCAACACAATACCACCTGAG-3’  Reverse: 5’-TCTCTGGTCCAGAGTGCAAGCT-3’ |
| *Ifnb1* | murine | Forward: 5’-GCAACCACCACTCATTCTGA-3’  Reverse: 5’-ATAAGCAGCTCCAGCTCCAA-3’ |
| *Ifna1* | murine | Forward: 5’- GGATGTGACCTTCCTCAGACTC-3’  Reverse: 5’-ACCTTCTCCTGCGGGAATCCAA-3’ |
| *Mda5 (Ifih1)* | murine | Forward: 5’-GCCTGGAACGTAGACGACAT-3’  Reverse: 5’-TGGTTGGGCCACTTCCATTT-3’ |
| *MMTV-Spliced* | murine | Forward: 5’-AGAGCGGAACGGACTCACCA-3′  Reverse: 5’-TCAGTGAAAGGTCGGATGAA-3′ |
| *GLN* | murine | Forward: 5’-TGTGTAAGTCCAGACGCAG-3′  Reverse: 5’-CCAACCTACTCCAAAAACAG-3′ |
| *IAP* | murine | Forward: 5’-AAGCAGCAATCACCCACTTTGG-3′  Reverse: 5’--CAATCATTAGATGCGGCTGCCAAG-3′ |
| *MMERVK* | murine | Forward: 5’-CAAATAGCCCTACCATATGTCAG-3′  Reverse: 5’-GTATACTTTCTTCTTCAGGTCCAC-3′ |
| *MusD* | murine | Forward: 5’-GTGCTAACCCAACGCTGGTTC-3′  Reverse: 5’-CTCTGGCCTGAAACAACTCCTG-3′ |
| *EntII* | murine | Forward: 5’-GTGCTAACCCAACGCTGGTTC-3′  Reverse: 5’-ACTGGGGCAATCCGCCTATTC-3′ |
| *Mervl Pol* | murine | Forward: 5’-ATCTCCTGGCACCTGGTATG-3  Reverse: 5’-AGAAGAAGGCATTTGCCAGA-3′ |
| *xMLV* | murine | Forward: 5’-TCTATGGTACCTGGGGCTC-3′  Reverse: 5’-GGCAGAGGTATGGTTGGAGTAG-3′ |
| *pMLV* | murine | Forward: 5’-CCGCCAGGTCCTCAATATAG-3  Reverse: 5’-AGAAGGTGGGGCAGTCT-3′ |
| *mpMLV* | murine | Forward: 5’-CCGCCAGGTCCTCAATATAG-3  Reverse: 5’-CGTCCCAGGTTGATAGAGG-3′ |
| *eMLV-Spliced* | murine | Forward: 5’-CCAGGGACCACCGACCCACCG-3’  Reverse: 5’--TAGTCGGTCCCGGTAGGCCTCG-3′ |
| *Actb (β-actin)* | Human | Forward: 5’-CACCATTGGCAATGAGCGGTTC-3’ Reverse: 5’-AGGTCTTTGCGGATGTCCACGT-3’ |
| *RIGI (DDX58)* | Human | Forward: 5’-CACCTCAGTTGCTGATGAAGGC-3’  Reverse: 5’-GTCAGAAGGAAGCACTTGCTACC-3’ |
| *MAVS* | Human | Forward: 5’-ATGGTGCTCACCAAGGTGTCTG-3’  Reverse: 5’-TCTCAGAGCTGCTGTCTAGCCA-3’ |
| *IFNB1* | Human | Forward: 5’-CTTGGATTCCTACAAAGAAGCAGC-3’  Reverse: 5’-TCCTCCTTCTGGAACTGCTGCA-3’ |
| *IFNA1* | Human | Forward: 5’-AGAAGGCTCCAGCCATCTCTGT-3’  Reverse: 5’-TGCTGGTAGAGTTCGGTGCAGA-3’ |
| *MDA5 (IFIH1)* | Human | Forward: 5’-GCTGAAGTAGGAGTCAAAGCCC-3’  Reverse: 5’-CCACTGTGGTAGCGATAAGCAG-3’ |
| *ERV-V2* | Human | Forward: 5’-CTTCTTTCTGAGCTCCTGTCTC-3’  Reverse: 5’-GTCCTCTGGTTCTTGGTCTTC-3’ |
| *ERVW1* | Human | Forward: 5’-CAAGTCCCTTCCCTCTAATTCC-3’  Reverse: 5’-TCCACTCCAGCCACTTTAAC-3’ |
| *ERV9* | Human | Forward: 5’-TCTTGGAGTCCTCACTCAAACTC-3’  Reverse: 5’-ACTGCTGCAACTACCCTTAAACA-3’ |
| *ERVK-1* | Human | Forward: 5’-ATCCTATGGCACCACCTAGTA-3’  Reverse: 5’-GCCTCAGTATCTCCTTCCTTTC-3’ |
| *ERV-H1* | Human | Forward: 5’-GCCCATTCTCTCTCTCCATATC-3’  Reverse: 5’-CCTGACATTCCTGCCTTCTTA-3’ |
| *ERVFXA34* | Human | Forward: 5’-CAGGAAACTAACTTTCAGCCAGA-3’  Reverse: 5’-TAAAGAGGGCATGGAGTAATTGA-3’ |
| *ERV-Fc2* | Human | Forward: 5’-CTGGAAGCTACACACTCCATAC-3’  Reverse: 5’-TGCCAAGAGGTGGGTTATTC-3’ |
| *ERV-FRD1* | Human | Forward: 5’-AGCCAGCTCTCAAAGGAAATAG-3’  Reverse: 5’-GAAGGACTACGGCTGCTAAAG-3’ |
| *ERVW2* | Human | Forward: 5’-CCACTGTCTGTTGGACTTACTT-3’  Reverse: 5’-TGGGAGATTGCTTCCTTTACTT-3’ |
| *ERV-Fb1* | Human | Forward: 5’-ATATCCCTCACCACGATCCTAATA-3’  Reverse: 5’-CCCTCTGTAGTGCAAAGACTGATA-3’ |
| *ERV-K10* | Human | Forward: 5’-GTCCCAAGTGTTTCAGGGAATA-3’  Reverse: 5’-GAAGCAGAGAGACTGCTTGTATAG-3’ |

**Supplementary Table 6: Western Blot Antibodies**

| **Antibody** | **Isotype** | **Clone** | **Vendor** | **Catalogue Number** |
| --- | --- | --- | --- | --- |
| Anti-rabbit IgG, HRP-linked Antibody | Goat IgG | Polyclonal | Cell Signaling | 7074S |
| RIG-I | Rabbit IgG | D14G6 | Cell Signaling | 3743S |
| MDA5 | Rabbit IgG | D74E4 | Cell Signaling | 5321S |
| MAVS (murine) | Rabbit IgG | - | Cell Signaling | 4983S |
| MAVS (human) | Rabbit IgG | D5A9E | Cell Signaling | 24930S |
| pSTAT1 | Rabbit IgG | 58D6 | Cell Signaling | 9167S |
| pIRF3 (murine) | Rabbit IgG | E6F7Q | Cell Signaling | 79945T |
| pIRF3 (human) | Rabbit IgG | E7J8G | Cell Signaling | 37829T |
| pNF-Κb-p65 | Rabbit IgG | 93H1 | Cell Signaling | 3033S |
| STAT1 | Rabbit IgG | - | Cell Signaling | 9172S |
| IRF3 | Rabbit IgG | D83B9 | Cell Signaling | 4302S |
| NF-Κb-p65 | Rabbit IgG | C22B4 | Cell Signaling | 4764S |
| DNMT1 | Rabbit IgG | D63A6 | Cell Signaling | 5032S |
| DNMT3A | Rabbit IgG | D23G1 | Cell Signaling | 3598S |
| β-Actin | Rabbit IgG | 13E5 | Cell Signaling | 4970S |

**Supplementary Table 7: Patient demographics and treatment characteristics for ERV Methylation Analysis (GSE152710) performed by Infinium Human Methylation 450K BeadChip from Illumina.**

| **Patient No.** | **Sample No. _Methylation** | **Disease Timepoint** | **Cytological Category Group** | **Sample Stage** |
| --- | --- | --- | --- | --- |
| **6** | 4R  6R | Diagnosis  9m | AML | Diagnosis  Response |
| **10** | 9R  10R | Diagnosis  3m | MDS | Diagnosis  Partial Response |
| **13** | 13R  14R | Diagnosis  6m | MDS | Diagnosis  Progression |
| **14** | 15R  16R | Diagnosis  6m | MDS | Diagnosis  No Response |
| **15** | 17R  19R | Diagnosis  24m | AML | Diagnosis  Partial Response |
| **16** | 20R  22R | Diagnosis  12m | AML | Diagnosis  No Response |
| **21** | 26R  28R | Diagnosis  6m | MDS | Diagnosis  No Response |
| **25** | 31R  33R | Diagnosis  6m | MDS | Diagnosis  Progression |
| **31** | 34R  35R | Diagnosis  3m | MDS | Diagnosis  Partial Response |
| **32** | 37R  39R | Diagnosis  5m | MDS | Diagnosis  Progression |
| **41** | 51R  53R | Diagnosis  6m | MDS | Diagnosis  Complete Response |
| **44** | 54R  58R | Diagnosis  12m | MDS | Diagnosis  Partial Response |
| **45** | 59R  61R | Diagnosis  6m | MDS | Diagnosis  Progression |
| **48** | 62R  63R | Diagnosis  3m | MDS | Diagnosis  No Response |
| **49** | 64R  65R | Diagnosis  3m | MDS | Diagnosis  Partial Response |
| **54** | 75R  77R | Diagnosis  12m | MDS | Diagnosis  Progression |
| **57** | 80R  83R | Diagnosis  16m | MDS | Diagnosis  Progression |
| **64** | 95R  98R | Diagnosis  12m | MDS | Diagnosis  Progression |
| **65** | 100R  101R | Diagnosis  12m | MDS | Diagnosis  Complete Response |
| **71** | 106R  108R | Diagnosis  6m | MDS | Diagnosis  Partial Response |
| **80** | 119R  121R | Diagnosis  12m | AML | Diagnosis  Progression |
| **82** | 125R  126R | Diagnosis  3m | MDS | Diagnosis  Partial Response |

**Complete response:** Bone marrow <5% myeloblasts with normal maturation of all cell lines.

**Partial response:** Bone marrow blasts decreased by ≥ 50% over pre-treatment but still >5%

blasts.

**No response:** Bone marrow >5% without a decrease by 50% over pre-treatment.

**Progression:** For patients with:

Less than 5% blasts: ≥ 50% increase in blasts to > 5% blasts

5%-10% blasts: ≥ 50% increase to > 10% blasts

10%-20% blasts: ≥ 50% increase to > 20% blasts

20%-30% blasts: ≥ 50% increase to > 30% blasts

**SUPPLEMENTARY FIGURE LEGENDS**

**Fig. S1: Confirmation of MAVS and STING genetic deletion in murine AML cells.**

Western blot imaging of MAVS and STING protein expression in AML^C1498^ (upper blots) and AML^WEHI-3B^ (lower blots) murine leukemia cells with WT and knockout (*Mavs^⁻/⁻^* or *Sting^⁻/⁻^)* backgrounds. β-Actin was used as a loading control. All proteins were detected on the same membrane; non-adjacent lanes were cropped and rearranged for clarity in presentation.

**Fig. S2: Immunophenotyping strategy of AML^C1498^ *in vivo.***

(A) Gating strategy for flow cytometric identification of leukemic cells in BM and spleen of AML^C1498^-bearing mice. (B) Representative flow cytometry plots showing leukemic cell populations in the BM and spleen of AML^C1498^-bearing mice, comparing WT and *Mavs^−/−^* groups with or without 5-AZA treatment.

**Fig. S3: 5-AZA does not significantly enhance STING expression in AML.**

(A) qPCR analysis of STING (TMEM173) expression in AML cell lines (c1498, WEHI-3B, MOLM-13) following DMSO or 5-AZA treatment. *p*-values were calculated using a two-sided unpaired non-parametric student’s t-test (Mann–Whitney) or parametric student’s t-test. (B) Representative western blots depicting STING protein levels upon 5-AZA treatment in AML^c1498^ and AML^MOLM-13^ cell lines.

**Fig. S4: Exposure to the HMA Decitabine induces expression of nucleic acid receptors signaling components in AML cells similar to 5-AZA.**

qPCR analysis of (A) nucleic acid receptor signaling components expression and (B) ERVs transcripts in C1498 and MOLM-13 AML cells following DMSO or 1μM DAC treatment for 7 days. Each grid on the heatmap denotes one technical replicate.

**Fig. S5: DNA-damaging chemotherapies suppress cellular growth of AML cells independent of intrinsic MAVS activity.**

Confluency growth assay of (A) AML^C1498^ and (B) AML^WEHI-3B^ murine WT and *Mavs*-deficient AML cell lines following exposure to DMSO or 1μM cytarabine (Ara-C), Daunorubicin, Idarubicin, and Fludarabine over 9 days. Data are presented as mean ± SEM from an experiment performed with technical triplicates.

**Fig. S6: 5-AZA-induced upregulation of RLHs in AML cells relies on MAVS-dependent feedback loops.**

qPCR analysis of RIG-I and MDA5 expression in *Mavs^-/-^* AML^C1498^ cells treated with DMSO or 1μM 5-AZA.

**Fig. S7: HMA-induced ERV or RLH mRNA upregulation is largely restricted to malignant cells and is not observed in (immune) cells in the spleen or BM of healthy mice.**

(A) qPCR analysis of ERVs transcripts in C1498 and MOLM-13 AML cell lines following *in vitro* DMSO or 1μM DAC treatment for 7 days. Each grid on the heatmap denotes one technical replicate. (B-E) C57BL/6j and BALB/c mice were treated with either DMSO in PBS or 2.5mg/kg 5-AZA in the absence of AML. qPCR analysis of known murine ERV transcripts in the spleen (top panels) and BM (bottom panels) of (B) C57BL/6j and (C) BALB/c mice. qPCR analysis of RLR signaling pathway receptors and signaling components in the spleen (top panels) and bone marrow (bottom panels) of steady state (D) C57BL/6j and (E) BALB/c mice following treatment with either PBS or 2.5 mg/kg 5-AZA. *p*-values were calculated using a two-sided unpaired non-parametric student’s t-test (Mann–Whitney).

**Fig. S8: Validation of RIG-I immunoprecipitation specificity.**

Representative western blot showing input protein from DMSO- or 5-AZA-treated AML^C1498^ cells immunoprecipitated with anti-RIG-I or polyclonal IgG, confirming RIG-I enrichment and specificity.

**Fig. S9: 5-AZA treatment enhances NK and CD8^+^ T cell activity in AML-bearing mice.**

AML^c1498^-bearing mice were treated with DMSO or 5-AZA. (A) Representative flow cytometry plots showing NK cell (NK1.1⁺NKG2D⁺NKp46⁺) frequency and activation marker expression (IFN-γ, TNF-α) in the BM (upper panels) and spleen (lower panels). (B-D) Quantification of NK cell responses in the spleen and BM: (B) Abundance of NK1.1⁺NKG2D⁺NKp46⁺ cells; (C) IFN-γ⁺ and (D) TNF-α⁺ expression levels on NK cells (fold change in median fluorescence intensity (MFI)). (E) Representative flow cytometry plots showing CD4^+^ and CD8⁺ T cell frequencies and IFN-γ expression in the BM and spleen. (F-I) Quantification of cell responses: (F) Abundance of CD3⁺CD4⁺ T cells; (G) IFN-γ expression levels on CD4⁺ T cells; (H) Abundance of CD3⁺CD8⁺ T cells; (I) IFN-γ expression levels on CD8⁺ T cells. Data are presented as mean ± SEM and pooled from two independent experiments.

**Fig. S10: 5-AZA-R AML^WEHI-3B^-bearing mice demonstrate no therapeutic response to 5-AZA.**

Kaplan Meier curve of overall survival in mice engrafted with AML^WEHI-3B^ comparing DMSO and 5-AZA treated groups of mice bearing either WT or 5-AZA-R AML backgrounds. Data were pooled from two independent experiments. *p*-values were calculated using the two-sided Mantel–Cox (log-rank) test.

**Fig. S11: UMAP Analysis Reveals Distinct Clustering of Healthy Controls and Overlapping Phenotypic Profiles between Treatment Responders and Non-Responders.**

Left panel: illustrates the low-dimensional embedding of the combined high-risk MDS and sAML cohort, capturing the broad phenotypic continuum of the disease spectrum. Right panel: sAML-only sub-cohort provide a high-resolution view of transcriptional or cellular profiles exclusive to acute transformation.

**Supplementary References**

1.	Müller U, Steinhoff U, Reis LFL, Hemmi S, Pavlovic J, Zinkernagel RM, et al. Functional Role of Type I and Type II Interferons in Antiviral Defense. Science. 1994 Jun 24;264(5167):1918–21. doi:10.1126/science.8009221

2.	Ghetti M, De Santis I, Tolomeo D, Ruggieri F, Bracci C, Bochicchio MT, et al. The molecular landscape of the C1498 murine acute myeloid leukemia cell line. Biomarker Research. 2026 Apr 10;14(1):40. doi:10.1186/s40364-026-00915-1
